# Synthesis of new functionalized thiazolo pyridine-fused and thiazolo pyridopyrimidine-fused spirooxindoles *via* one-pot reactions

**DOI:** 10.1016/j.heliyon.2020.e03687

**Published:** 2020-03-31

**Authors:** Shima Nasri, Mohammad Bayat, Hassan Vasheghani Farahani, Solmaz Karami

**Affiliations:** Department of Chemistry, Faculty of Science, Imam Khomeini International University, Qazvin, Iran

**Keywords:** Organic chemistry, Spiro heterocycles, Spirooxindoles, Nitroketene dithioacetals, Isatin, Nano-SiO_2_

## Abstract

A sequential multi-component reaction of the nitroketene dithioacetals, cysteamine hydrochloride, isatin and different CH-acids is described. This efficient method provides new functionalized thiazolo pyridine-fused spirooxindoles and thiazolo pyridopyrimidine-fused spirooxindoles in good yields. In the case of using isatin derivatives (5-bromoisatin and 5-chloroisatin), the reaction was carried out by using nano-SiO_2_ (20 mol%) as an effective heterogeneous Lewis acid promoter. This type of reaction provides a range of skeletally different polycyclic spiro thiazole-based heterocyclic structures and represents attractive advantages including straightforward one-pot operation under the catalyst-free condition and simple workup procedures without using tedious purification procedure.

## 1 Introduction

Spiroheterocycles moieties frequently observed in central skeletons of numerous bioactive natural alkaloids and due to its high medicinal properties, occupied a specific region in the heterocyclic field, and also they have become valuable therapeutic targets in drug discovery layouts because of inherent three-dimensional character and capability to functionalities in all three dimensions [1, 2]. The existence of spiro center in molecules leads to structural rigidity and complexity and subsequently increasing its dependency on proteins [3].

**Figure 1:**
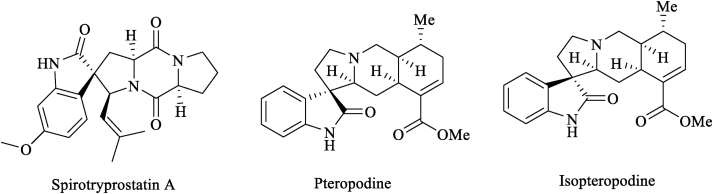
A representative of spirooxindole containing drugs.

Spirooxindoles are known as a subclass of indole that 3-carbon position of indole sharing in the constitution of spiroindole structures which makes them as core building blocks of many synthetic drugs [4, 5], and natural functionalized organic compounds which raise their biological properties [6, 7]. Spirooxindoles are valuable synthetic targets in organic chemistry and pharmacological research fields due to their remarkable biological properties including antimicrobial [8], antitumor [9], antidiabetic [10], potential antileukemic, local anesthetic [11], antifungal activities [12] and can also use as intermediates in synthetic steps for many types of medicinal precursors [13], e.g. spirotryprostatin A exhibit microtubule assembly inhibition while isopteropodine and pteropodine adjust the action of muscarinic serotonin receptors (Figure 1) [5,11]. Numerous reactions have been designed for the formation of diverse recognized spiro-based heterocycles by the classical cyclocondensation procedure [14, 15, 16].

**Figure 2:**
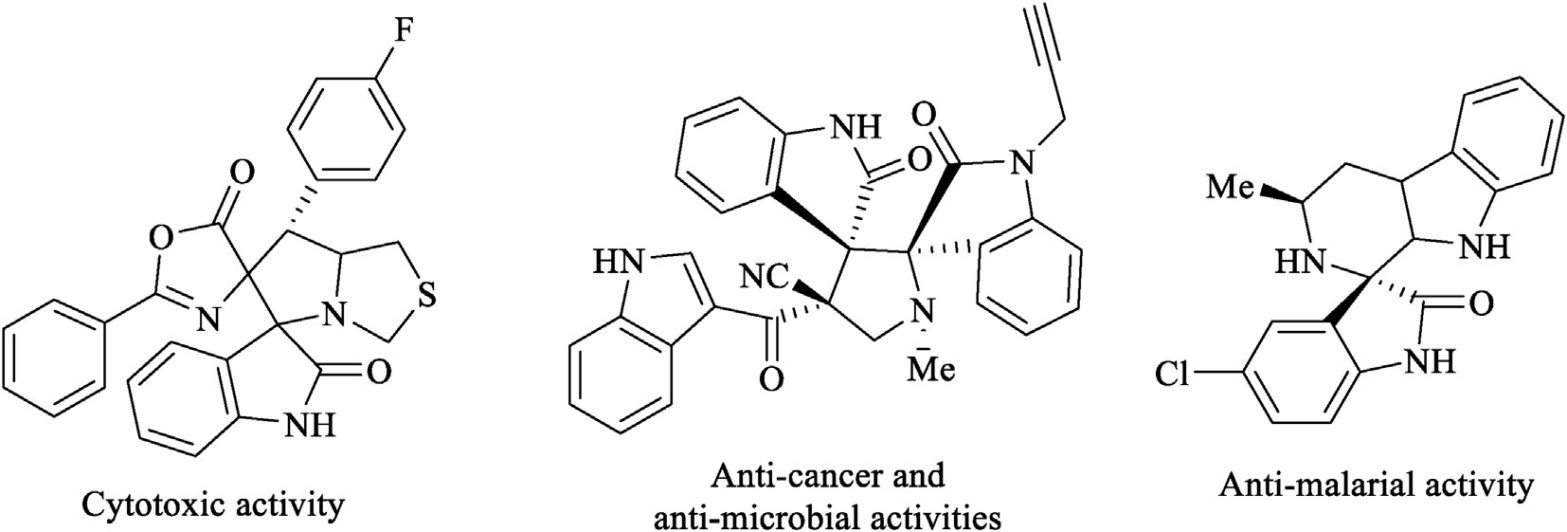
Selected representative biologically active polycyclic spiro-based compounds.

In spiro heterocyclic structures, the existence of the two or more various heterocyclic organic frameworks in a single molecule could significantly increase biological activity and also be considered as models for drug designing (Figure 2) [1,17]. The functionalization and changing of R-groups located on the spiro compounds are effectively expanded the degree of structural diversity within a class of compounds and subsequently increased the possibility of arriving at the wide or distinguished biological activity [18].

**Scheme 1:**
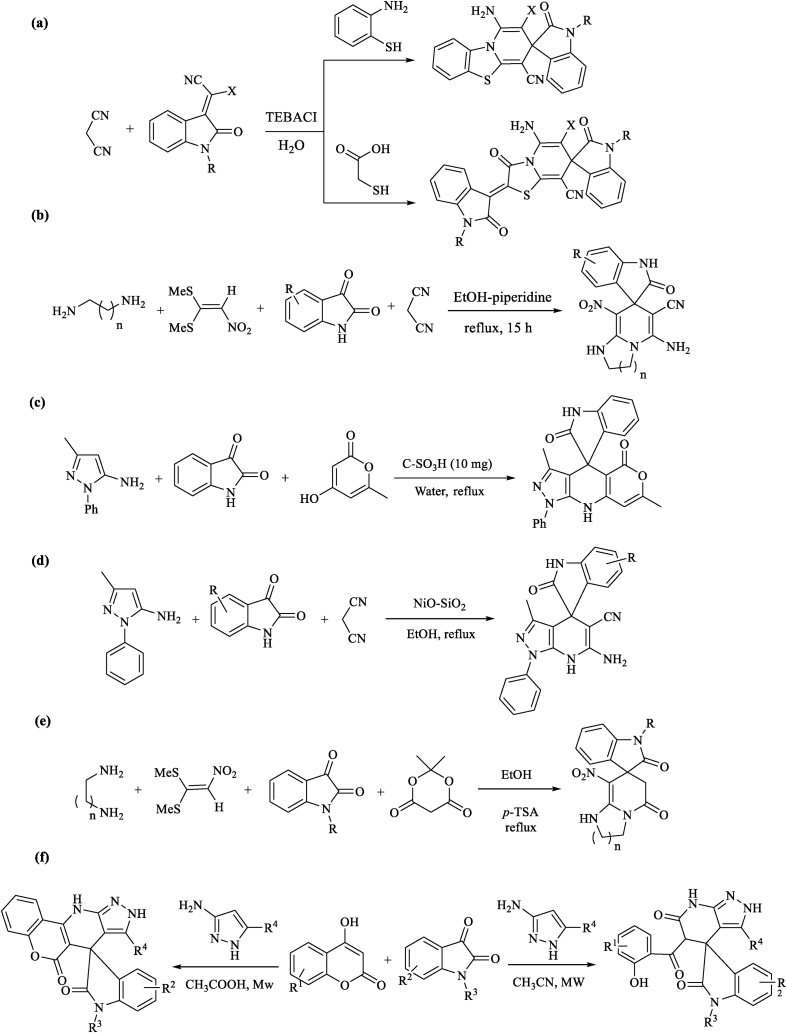
Summary of previous studies for the synthesis of pyridine-fused spirooxindoles.

A series of methods have been reported for the synthesis of these structures with three-, four-, five-, or six-membered rings fused at the shared carbon location with therapeutic applications to treat many human diseases and cancers [6, 15]. However, the development of an easy, efficient and catalyst-free synthetic approach to the preparation of spiro thiazole-based heterocycles still remains an essential need, nearly due to the existence of obstacles in the formation of polycyclic heterocycles including spiro-quaternary stereocenters. Because of the biological importance of pyridine-fused spirooxindoles, numerous methods have been reported for the synthesis of these structures, e.g. in 2012, Hussein et al. have reported an easy and efficient one-pot three-component method for the synthesis of spiro{pyrido[2,1-*b*]benzothiazole-3,3′-indoline} and/or spiro{thiazolo[3,2-*a*]pyridine-7,3′-indoline} products by the addition of 2-mercaptoaniline and/or mercaptoacetic acid, malononitrile, and a group of 2-oxoindoline-3-ylidines in water (Scheme 1a) [19]. In 2010, Alizadeh et al. have described an analogous MCR to prepare the structurally similar spiro imidazole containing heterocycles starting from 1,1-bis(methylthio)-2-nitroethylene, 1,n-diamine, isatin, or its derivatives, and malononitrile in EtOH using piperidine as a basic catalyst (10 mol%) undergo heating at reflux temperature (Scheme 1b) [20]. In 2018, Li et al. have established the synthesis of spirooxindole fused pyrazolopyridine productes *via* a fast and efficient three-component domino reaction by using 4-hydroxy-6-methyl-2*H*-pyran-2-one, 3-methyl-1-phenyl-1*H*-pyrazol-5-amine, and isatin in a green process (Scheme 1c). In 2018, Yagnam and coworkers have described a one-pot three-component reaction of 5-amino-3-methylpyrazole, isatin, and malononitrile for the synthesis of spirooxindole-fused pyrazolo pyridine derivatives in the presence of NiO–SiO_2_ catalyst (Scheme 1d). In 2019, Rahimi et al. have developed a four-component reaction of 1,1-bis(methylthio)-2-nitroethylene, different diamines, isatin derivatives, and Meldrum's acid using *p*-toluenesulfonic acid as a solid acid catalyst for the synthesis of new spiropyridineoxindole derivatives containing pyridone moiety (Scheme 1e). In 2019, Mishra and coworkers have reported the synthesis of two various kinds of pyridine-fused spirooxindoles *via* a three-component reaction of isatin, 4-hydroxycoumarin and aminopyrazole/aminoisoxazole under microwave irradiation conditions which this reaction is highly dependent on the reaction medium (Scheme 1f). Although the previous literature show the formation of the compounds with analogous structures, designing novel practical, fine, efficient and catalyst-free synthetic methods for these new structurally skeleton spiro thiazole-based heterocycles is highly desirable.

General interest in spiro thiazole-based heterocycles structures comes not only from their structural properties but also from their biological applications, so introducing new synthetic procedures for the synthesis of spiro thiazole-based heterocycles has been an active field of chemical research for well over a century and would be beneficial to develop new therapeutic agents [21, 22, 23]. In continuation of our work on the expansion of new methods to construct potential biologically active heterocycles [24, 25, 26] and considering typical substituent impacts on bioactive properties, we herein report new synthetic approaches to sulfur-containing spiro heterocycle molecules. For example diverse thiazolo pyridine-fused spirooxindoles and thiazolo pyridopyrimidine-fused spirooxindoles as attractive synthetic targets designed and synthesized. The chemical structures identified by spectroscopic methods. The products generally containing unique sulfur atom and were acquired in good efficiencies, with simple workup processes and easy isolation.

## 2 Experimental

### 2.1 Reagent and apparatus

The nitroketene dithioacetals, cysteamine hydrochloride, isatin, malononitrile, ethyl cyanoacetate, methyl cyanoacetate, cyanoacetohydrazide, barbituric acid, derivatives of isatin, triethylamine and solvents were obtained from Sigma Aldrich and used without further purification. Nano-silica (CAB-O-SIL® M5) was obtained from Cabot Co. IR spectra: Bruker Tensor 27 spectrometer. NMR spectra: Bruker DRX-300 Avance instrument (300 MHz for ^1^H and 75.4 MHz for ^13^C) with DMSO-*d*_6_ as solvents. Chemical shifts are expressed in parts per million (ppm), and coupling constant (*J*) are reported in hertz (Hz). Mass spectra: Agilent 5975C VL MSD with Triple-Axis detector operating at an ionization potential of 70 eV. Elemental analyses for C, H and N: Heraeus CHNO-Rapid analyzer. Melting points: electrothermal 9100 apparatus.

### 2.2 General procedure for the synthesis of 5a-c, 5h and 6a

A mixture of cysteamine hydrochloride (0.113 g, 1 mmol), 1,1- bis(methylthio)-2-nitro ethylene (0.165 g, 1 mmol), triethylamine (139 μL, 1 mmol), and 10 mL EtOH in a 50 mL round-bottomed flask equipped with a reflux condenser, was heated with stirring in an oil-bath at reflux temperature for 4 h, after that isatin or its derivatives (1 mmol) and CH-acid compound (1 mmol) were added to the reaction medium, and it was stirred under 80 °C for a defined time in Figures 4 and 5, which controlled by TLC, ethyl acetate/n-hexane, 6:4. Then, the temperature of the reaction mixture decreased to room temperature and the precipitate filtered to obtain the product. The crude product **5** acquires by doing the washing with 96% ethanol and drying in the oven in 150 °C on precipitate. The obtained product recrystallized by using ethanol to provide the pure product (for CHN analyses).

### 2.3 General procedure for the synthesis of 5d-g, and 6b

This method is similar to the previous one, but only nano-SiO_2_ (20 mol%) was added to the mixture. In order to recycle the nanoparticles, the precipitate was extracted with DMF (10 mL), and the nano-SiO_2_ was recycled by washing with ethanol and drying after filtering. Finally, the DMF was evaporated under reduced pressure.

### 2.4 Spectral data

#### 2.4.1 5′-Amino-8′-nitro-2-oxo-2′,3′-dihydrospiro[indoline-3,7′-thiazolo[3,2-a]pyridine]-6′-carbonitrile (5a)

Yellow powder: mp = 329 °C, 0.289 g, yield 85%; IR (KBr) (ν_max_/cm^−1^): 3347 and 3241 (NH, NH_2_), 2189 (C≡N), 1712 (C=O), 1610 and 1448 (NO_2_), 1252 (C–N), 1133 (C–O), 761 (Ar). ^1^H NMR (300 MHz, DMSO-*d*_*6*_): *δ* 3.38–3.49 (2H, m, CH_2_S), 4.21–4.37 (2H, m, CH_2_N), 6.72 (2H, s, NH_2_), 6.78 (1H, d, ^3^*J*_HH_ = 8.1 Hz, ArH), 6.89–6.94 (1H, d, ^3^*J*_HH_ = 7.2 Hz, ArH), 7.14–7.18 (2H, m, ArH), 10.49 (H, s, NH); ^13^CNMR (75 MHz, DMSO-*d*_*6*_): *δ* 28.2 (CH_2_S), 51.9 (CH_2_N), 52.2 (C_spiro_), 62.2 (C–CN), 109.8 (C–NO_2_), 118.5 (CN), 120.8, 122.4, 123.8, 129.1, 133.6, 142.0 (Ar), 150.0 (S–C–N), 160.2 (N–C–N), 177.5 (C=O); MS (EI, 70 eV): *m/z* (%) = 341 (12) [M]^+^, 265 (100). Anal. Calcd for C_15_H_11_N_5_O_3_S (341.06): C, 52.78; H, 3.25; N, 20.52. Found C, 52.31; H, 2.97; N, 20.82.

#### 2.4.2 Ethyl-5′-amino-8′-nitro-2-oxo-2′,3′-dihydrospiro[indoline-3,7′-thiazolo[3,2-a]pyridine]-6′ carboxylate (5b)

Yellow powder: mp = 283 °C, 0.271 g, yield 70%; IR (KBr) (ν_max_/cm^−1^): 3343 (NH), 1705 (C=O), 1667 (C=O), 1568 and 1459 (NO_2_), 1253 (C–N), 1149 (C–O), 757 (Ar). ^1^H NMR (300 MHz, DMSO-*d*_*6*_): *δ* 0.76–0.81 (3H, t, ^3^*J*_HH_ = 7.2 Hz, CH_3_), 3.38–3.41 (2H, q, ^3^*J*_HH_ = 7.2 Hz, CH_2_), 3.65–3.72 (2H, m, CH_2_S), 4.30–4.35 (2H, m, CH_2_N), 6.65 (1H, d, ^3^*J*_HH_ = 7.5 Hz, ArH), 6.76–6.81 (1H, t, ^3^*J*_HH_ = 7.2 Hz, ArH), 7.00–7.04 (1H, d, ^3^*J*_HH_ = 7.2 Hz, ArH), 7.07 (1H, d, ^3^*J*_HH_ = 7.5 Hz, ArH), 8.08 (2H, br s, NH_2_), 10.24 (H, s, NH); ^13^CNMR (75 MHz, DMSO-*d*_*6*_): *δ* 13.5 (CH_3_), 27.6 (CH_2_S), 51.6 (CH_2_N), 52.2 (C_spiro_), 56.5 (CH_2_), 80.7 (C–CO_2_Et), 108.5 (C–NO_2_), 121.3, 123.3, 123.9, 128.2, 134.5, 144.4 (Ar), 151.4 (S–C–N), 159.1 (N–C–N), 168.4 (CO_2_), 179.5 (C=O); MS (EI, 70 eV): *m/z* (%) = 388 (20) [M]^+^, 315 (100). Anal. Calcd for C_17_H_16_N_4_O_5_S (388.08): C, 52.57; H, 4.15; N, 14.43. Found C, 52.45; H, 3.87; N, 13.99.

#### 2.4.3 Methyl-5′-amino-8′-nitro-2-oxo-2′,3′-dihydrospiro[indoline-3,7′-thiazolo[3,2-a]pyridine]-6′-carboxylate (5c)

Yellow powder: mp = 275 °C, 0.284 g, yield 76 %; ^1^H NMR (300 MHz, DMSO-*d*_*6*_): *δ* 3.20 (3H, s, CH_3_), 3.36–3.38 (2H, m, CH_2_), 4.30–4.35 (2H, m, CH_2_), 6.67 (1H, d, ^3^*J*_HH_ = 7.5 Hz, ArH), 6.76–6.81 (1H, t, ^3^*J*_HH_ = 7.5 Hz, ArH), 7.01–7.08 (2H, m, ArH), 8.03 (2H, s, NH_2_), 10.24 (H, s, NH); ^13^CNMR (75 MHz, DMSO-*d*_*6*_): *δ* 27.6 (CH_2_S), 50.4 (CH_3_), 51.7 (CH_2_N), 52.2 (C_spiro_), 80.9 (C–CO_2_Me), 108.5 (C–NO_2_), 121.4, 123.3, 123.9, 128.3 134.4, 144.1 (Ar), 151.3 (S–C–N), 159.1 (N–C–N), 168.6 (CO_2_), 179.6 (C=O); MS (EI, 70 eV): *m/z* (%) = 374 (26) [M]^+^, 59 (100). Anal. Calcd for C_16_H_14_N_4_O5_4_S (374.07): C, 51.33; H, 3.77; N, 14.97. Found C, 51.15; H, 3.43; N, 15.32.

#### 2.4.4 5′-Amino-4-bromo-8′-nitro-2-oxo-2′,3′-dihydrospiro[indoline-3,7′-thiazolo[3,2-a]pyridine]-6′-carbonitrile (5d)

Yellow powder: mp = 263 °C, 0.315 g, yield 75 %; ^1^H NMR (300 MHz, DMSO-*d*_*6*_): *δ* 4.17–4.25 (2H, m, CH_2_S), 4.31–4.40 (2H, m, CH_2_N), 6.75 (1H, d, ^3^*J*_HH_ = 8.4 Hz, ArH), 6.81 (2H, s, NH_2_), 7.33 (1H, d, ^3^*J*_HH_ = 8.4 Hz, ArH), 7.49 (1H, s, ArH), 10.64 (H, s, NH); ^13^CNMR (75 MHz, DMSO-*d*_*6*_): *δ* 28.3 (CH_2_S), 51.9 (CH_2_N), 52.4 (C_spiro_), 61.5 (C–CN), 111.7 (C–NO_2_), 114.2 (CN), 118.5, 120.2, 126.8, 131.8, 136.0, 141.4 (Ar), 150.1 (S–C–N), 160.8 (N–C–N), 177.2 (C=O); MS (EI, 70 eV): *m/z* (%) = 418 (2) [M]^+^, 45 (100). Anal. Calcd for C_15_H_10_BrN_5_O_3_S (418.97): C, 42.87; H, 2.40; N, 16.67. Found C, 42.64; H, 2.05; N, 17.01.

#### 2.4.5 Ethyl-5′-amino-4-bromo-8′-nitro-2-oxo-2′,3′-dihydrospiro[indoline-3,7′-thiazolo[3,2-a]pyridine]-6′-carboxylate (5e)

Yellow powder: mp = 266 °C, 0.307 g, yield 66 %; IR (KBr) (ν_max_/cm^−1^): 3320 (NH), 1706(C=O), 1628 (C=O), 1575 and 1460 (NO_2_), 1252 (C–N), 1156 (C–O), 769 (Ar). ^1^H NMR (300 MHz, DMSO-*d*_*6*_): *δ* 0.72–0.86 (3H, t, ^3^*J*_HH_ = 7.2 Hz, CH_3_), 3.12–3.46 (2H, q, ^3^*J*_HH_ = 6.9 Hz, CH_2_), 3.68–3.75 (2H, m, CH_2_S), 4.24–4.39 (2H, m, CH_2_N), 6.62 (1H, d, ^3^*J*_HH_ = 8.1 Hz, ArH), 7.24 (1H, d, ^3^*J*_HH_ = 8.1 Hz, ArH), 7.31 (1H, s, ArH), 8.13 (2H, br s, NH_2_), 10.40 (H, s, NH); ^13^CNMR (75 MHz, DMSO-*d*_*6*_): *δ* 13.5 (CH_3_), 27.6 (CH_2_S), 51.7 (CH_2_N), 52.4 (C_spiro_), 56.5 (CH_2_), 80.1 (C–CO_2_Et), 110.4 (C–NO_2_), 113.0, 123.2, 126.3, 130.8, 137.0, 143.8, (Ar), 151.5 (S–C–N), 159.7 (N–C–N), 168.2 (CO_2_), 179.2 (C=O); MS (EI, 70 eV): *m/z* (%) = 465.9 (2) [M]^+^, 60 (100). Anal. Calcd for C_17_H_15_BrN_4_O_5_S (465.99): C, 43.70; H, 3.24; N, 11.99. Found C, 43.99; H, 3.57; N, 12.17.

#### 2.4.6 5′-Amino-6-chloro-8′-nitro-2-oxo-2′,3′-dihydrospiro[indoline-3,7′-thiazolo[3,2-a]pyridine]-6′-carbonitrile (5f)

Yellow powder: mp = 333 °C, 0.243 g, yield 65%; IR (KBr) (ν_max_/cm^−1^): 3350 and 3238 (NH, NH_2_), 2152 (C≡N), 1701 (C=O), 1592 and 1388 (NO_2_), 1250 (C–N), 1115 (C–O), 586 (C–Cl). ^1^H NMR (300 MHz, DMSO-*d*_*6*_): *δ* 3.40–3.50 (2H, m, CH_2_S), 4.18–4.41 (2H, m, CH_2_N), 6.79 (1H, s, ArH), 6.82 (2H, s, NH_2_), 7.21–7.24 (1H, d, ^3^*J*_HH_ = 8.1 Hz, ArH), 7.39–7.40 (1H, d, ^3^*J*_HH_ = 8.1 Hz, ArH), 10.64 (H, s, NH); ^13^CNMR (75 MHz, DMSO-*d*_*6*_): *δ* 28.3 (CH_2_S), 51.9 (CH_2_N), 52.5 (C_spiro_), 61.4 (C–CN), 111.2 (C–NO_2_), 118.5 (CN), 120.2, 124.2, 126.4, 128.9, 135.6, 141.0 (Ar), 150.2 (S–C–N), 160.8 (N–C–N), 177.4 (C=O). Anal. Calcd for C_15_H_10_ClN_5_O_3_S (375.02): C, 47.94; H, 2.68; N, 18.64. Found C, 48.20; H, 2.51; N, 18.77.

#### 2.4.7 Ethyl-5′-amino-6-chloro-8′-nitro-2-oxo-2′,3′-dihydrospiro[indoline-3,7′-thiazolo[3,2-a]pyridine]-6′-carboxylate (5g)

Yellow powder: mp = 355 °C, 0.261 g, yield 62%. ^1^H NMR (300 MHz, DMSO-*d*_*6*_): *δ* 0.72–0.85 (3H, t, ^3^*J*_HH_ = 6.9 Hz, CH_3_), 3.42–3.47 (2H, q, ^3^*J*_HH_ = 6.9 Hz, CH_2_), 3.68–3.75 (2H, m, CH_2_S), 4.25–4.40 (2H, m, CH_2_N), 6.66 (1H, d, ^3^*J*_HH_ = 8.1 Hz, ArH), 7.11 (1H, d, ^3^*J*_HH_ = 7.2 Hz, ArH), 7.20 (1H, s, ArH), 8.13 (2H, br s, NH_2_), 10.40 (H, s, NH); ^13^CNMR (75 MHz, DMSO-*d*_*6*_): *δ* 13.5 (CH_3_), 27.6 (CH_2_S), 51.7 (CH_2_N), 52.4 (C_spiro_), 56.5 (CH_2_), 80.1 (C–CO_2_Et), 109.8 (C–NO_2_), 123.3, 123.7, 125.3, 127.9, 136.6, 143.4 (Ar), 151.5 (S–C–N), 159.7 (N–C–N), 168.2 (CO_2_), 179.4 (C=O). Anal. Calcd for C_17_H_15_ClN_4_O_5_S (422.05): C, 48.29; H, 3.58; N, 13.25. Found C, 48.00; H, 3.32; N, 12.94.

#### 2.4.8 5′-Amino-8′-nitro-2-oxo-2′,3′-dihydrospiro[indoline-3,7′-thiazolo[3,2-a]pyridine]-6′ carbohydrazide (5h)

Yellow powder: mp = 374 °C, 0.273 g, yield 73%; IR (KBr) (ν_max_/cm^−1^): 3349, 3295, 3258 (NH, NH_2_), 1705 (C=O), 1611 (C=O), 1471 and 1387 (NO_2_), 1230 (C–N), 1187 (C–O), 751 (Ar). ^1^H NMR (300 MHz, DMSO-*d*_*6*_): *δ* 4.05–4.48 (4H, m, CH_2_), 6.87 (1H, d, ^3^*J*_HH_ = 7.2 Hz, ArH), 6.92–7.12, 7.35–7.40 (3H, m, ArH), 8.08 (2H, br s, NH_2_), 10.83 (2H, s, NH_2_), 11.30 (H, br s, NH), 11.59 (H, s, NH); ^13^CNMR (75 MHz, DMSO-*d*_*6*_): *δ* 25.5 (CH_2_S), 51.5 (CH_2_N), 52.9 (C_spiro_), 80.5 (C–CON_2_H_3_), 111.2 (C–NO_2_), 115.4, 116.2, 122.2, 126.7, 133.4, 138.2 (Ar), 144.5, (S–C–N), 164.7 (N–C–N), 168.4 (CON_2_H_3_), 179.0 (C=O); MS (EI, 70 eV): *m/z* (%) = 374 (0.1) [M]^+^, 132 (100). Anal. Calcd for C_15_H_14_N_6_O_4_S (374.08): C, 48.12; H, 3.77; N, 22.45. Found C, 47.93; H, 3.47; N, 22.07.

#### 2.4.9 6′-Nitro-8′,9′-dihydrospiro[indoline-3,5′-thiazolo[3′,2':1,6]pyrido[2,3-d]pyrimidine]-2,2′,4′(1′H,3′H)-trione (6a)

Orange powder: mp = 332 °C, 0.327 g, yield 85%. ^1^H NMR (300 MHz, DMSO-*d*_*6*_): *δ* 3.41–3.46 (2H, m, CH_2_S), 4.35–4.51 (2H, m, CH_2_N), 6.68 (1H, d, ^3^*J*_HH_ = 7.5 Hz, ArH), 6.81 (1H, d, ^3^*J*_HH_ = 7.2 Hz, ArH), 7.06–7.14 (2H, m, ArH), 10.42, 11.00 (2H, s, NH, NH), 11.18 (H, s, NH); ^13^CNMR (75 MHz, DMSO-*d*_*6*_): *δ* 28.4 (CH_2_S), 51.0 (CH_2_N), 51.9 (C_spiro_), 92.7 (C=C–C=O), 108.8 (C–NO_2_), 121.4, 123.4, 123.7, 128.7, 132.3, 144.1 (Ar), 150.3 (S–C–N), 159.6 (N–C–N), 160.9 (C=O), 161.1 (C=O), 177.6 (C=O); MS (EI, 70 eV): *m/z* (%) = 385 (0.1) [M]^+^, 60 (100). Anal. Calcd for C_16_H_11_N_5_O_5_S (385.05): C, 49.87; H, 2.88; N, 18.17. Found C, 50.19; H, 3.05; N, 18.29.

#### 2.4.10 6-Chloro-6′-nitro-8′,9′-dihydrospiro[indoline-3,5′-thiazolo[3′,2':1,6]pyrido[2,3-d]pyrimidine]-2,2′,4′(1′H,3′H)-trione (6b)

Orange powder: mp = 335 °C, 0.318 g, yield 76%. ^1^H NMR (300 MHz, DMSO-*d*_*6*_): *δ* 3.37–3.43 (2H, m, CH_2_S), 4.29–4.55 (2H, m, CH_2_N), 6.68 (1H, d, ^3^*J*_HH_ = 8.1 Hz, ArH), 7.12 (1H, d, ^3^*J*_HH_ = 8.1 Hz, ArH), 7.82 (1H, s, ArH), 10.01, 10.97 (2H, s, NH, NH), 11.25 (H, s, NH); ^13^CNMR (75 MHz, DMSO-*d*_*6*_): *δ* 28.9 (CH_2_S), 50.6 (CH_2_N), 51.9 (C_spiro_), 95.1 (C=C–C=O), 109.0 (C–NO_2_), 120.2, 122.4, 123.5, 129.2, 132.4, 145.2 (Ar), 150.7 (S–C–N), 159.1 (N–C–N), 160.3 (C=O), 161.5 (C=O), 177.0 (C=O); MS (EI, 70 eV): *m/z* (%) = 419 (5) [M]^+^, 44 (100). Anal. Calcd for C_16_H_10_ClN_5_O_5_S (419.01): C, 45.78; H, 2.40; N, 16.68. Found C, 46.01; H, 2.11; N, 16.28.

## 3 Results and discussion

**Scheme 2:**
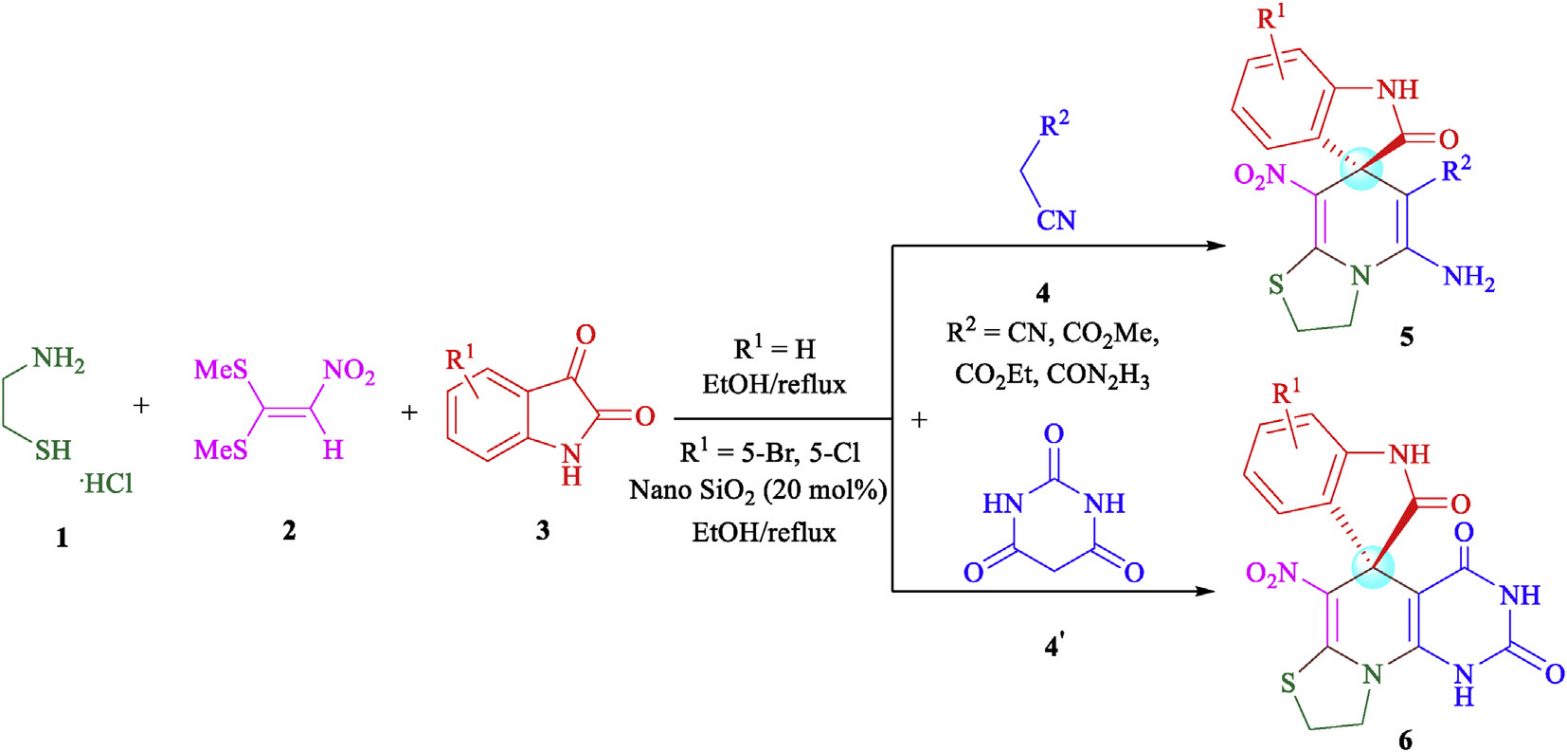
Synthetic approaches for the formation of products 5 and 6.

In this study, novel structures containing thiazolo pyridine-fused spirooxindoles **5** and thiazolo pyridopyrimidine-fused spirooxindoles **6** were synthesized by a four-component domino reaction of cysteamine hydrochloride **1**, nitroketene dithioacetals **2**, isatin or its derivatives **3** and active methylene compounds (malononitrile, ethyl cyanoacetate, methyl cyanoacetate, cyanoacetohydrazide and barbituric acid) **4** and **4′** in ethanol under reflux conditions (Scheme 2). In the case of using isatin derivatives (5-bromoisatin and 5-chloroisatin), due to the less activity of these derivatives than isatin, the reaction was carried out by using nano-SiO_2_ (20 mol%) as an effective heterogeneous Lewis acid promoter.

**Figure 3:**
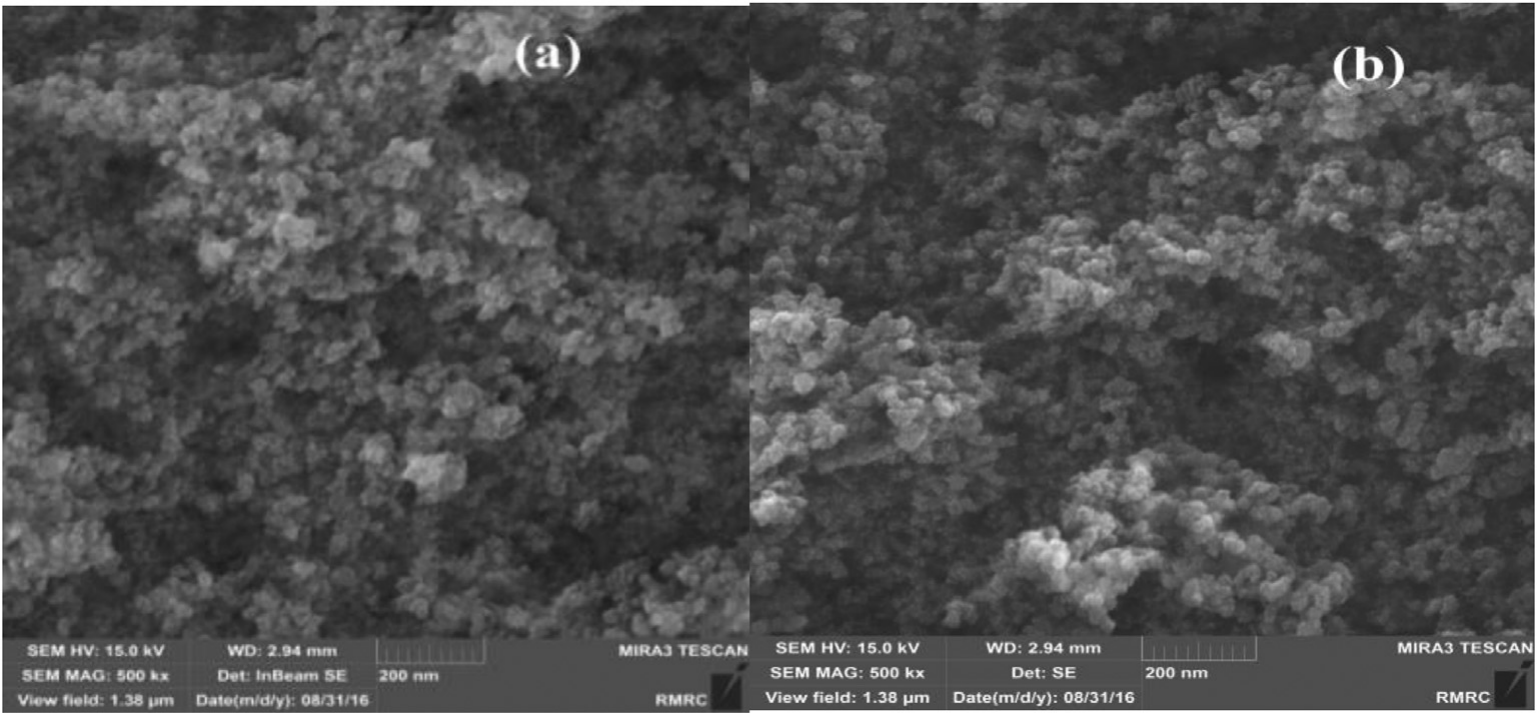
SEM image of nano-SiO_2_ (a) before and (b) after reuse.

The dimensions and porous structure of the SiO_2_ nanoparticles were also surveyed using scanning electron microscopy (SEM) (Figure 3a). Figure 3a demonstrates the range of nano dimensions is between 60 and 90 nm and the basic morphology of the particles was approximately spherical with smooth surfaces. Also, the SEM image shows an acceptable aggregation of silica particles. In the following, by keeping constant the reaction conditions, the activity of the recycled nano-SiO_2_ was also studied. The recycled nano-SiO_2_ was reused and product efficiency don't decrease significantly which this result confirmed by the SEM image of SiO_2_ nanoparticles after reuse (Figure 3b) that the characterization of the nano-SiO_2_ before and after reuse most nearly same in morphology and particle size.

**Scheme 3:**
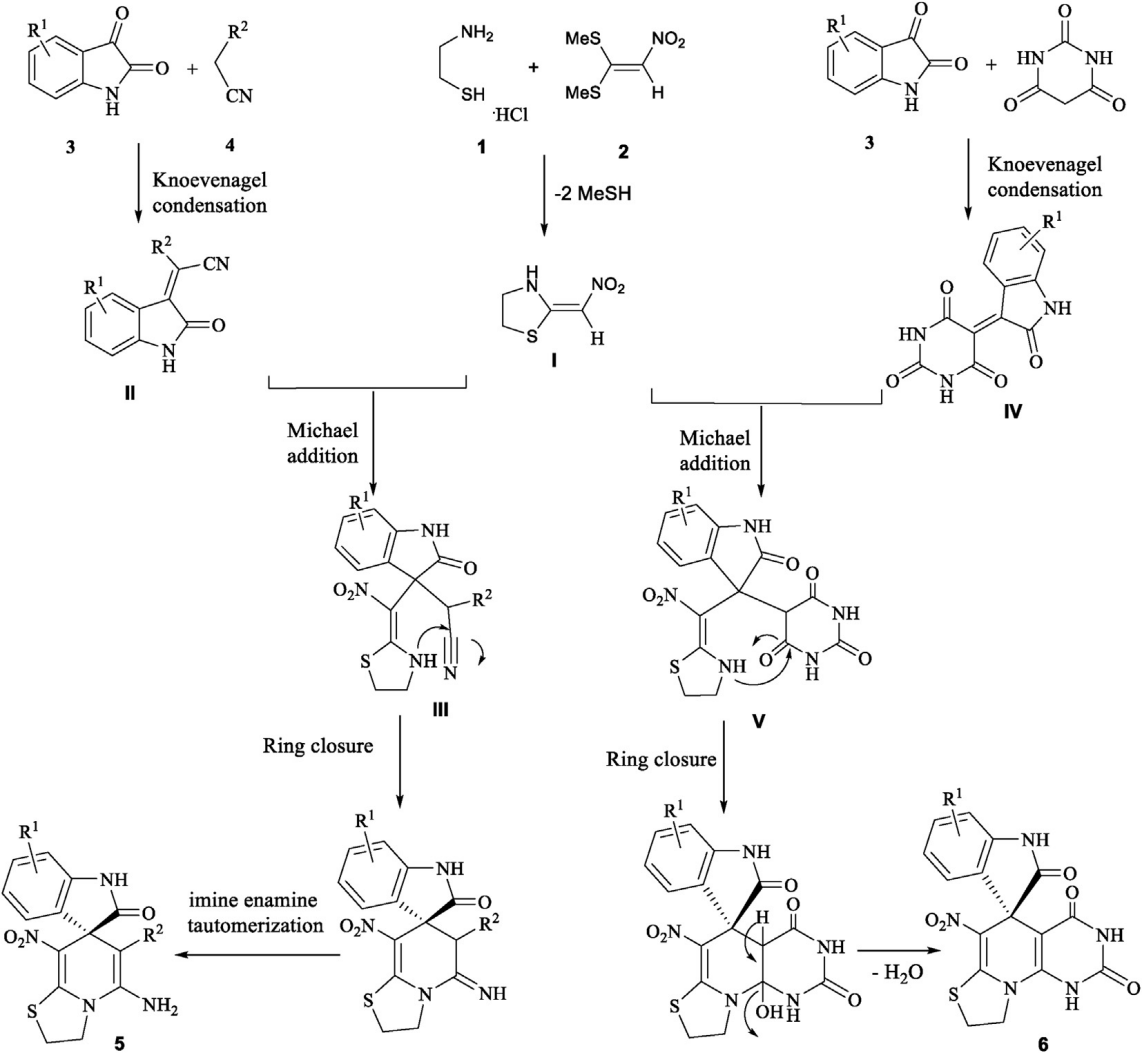
A plausible mechanism for the synthesis of 5 and 6.

On the basis of the chemistry of ketene *N,S*-acetals [27] and ketene *N,N*-acetals [20, 28], a possible mechanism [29] is proposed in Schemes 3. For the synthesis of product **5**, it is possible that at first, the creation of 2-nitromethylene thiazolidine **I** happens *via* the interaction between cysteamine hydrochloride **1** to 1,1-bis (methylthio)-2-nitroethene **2** by using an equivalent amount of triethylamine base in order to release of cysteamine salts. Then *Michael* acceptor **II** causes *via Knoevenagel* condensation between isatin **3** and malononitrile/ethyl cyanoacetate/methyl cyanoacetate/cyanoacetohydrazide **4** that followed by elimination of water molecules. The 2-nitromethylene thiazolidine **I** then attacks to the *Knoevenagel* product **II** in a *Michael* addition to provide open-chain intermediate **III**, that due to *N*-cyclization *via* attack of the secondary amino group to the nitrile functional group of **4**, followed by successive imine-enamine tautomerization give product **5.** While in the case of the synthesis of product **6**, the formation of these heterocycles can be followed through similar mechanism. Clearly, the reaction proceeds by condensation between barbituric acid **4′** and carbonyl group positioned on isatin **3** to obtain the *Knoevenagle* intermediate **IV**. In the following, this *Knoevenagle* intermediate undergoes *Michael*-type addition reaction by the attack of 2-nitromethylene thiazolidine **I** generates open chain intermediate **V**. Eventually, the nucleophilic addition of the amino group to the carbonyl group occurs, then dehydration and *N*-cyclization sequence lead to the formation of thiazolo pyridopyrimidine-fused spirooxindoles **6** (Scheme 3).

**Figure 4:**
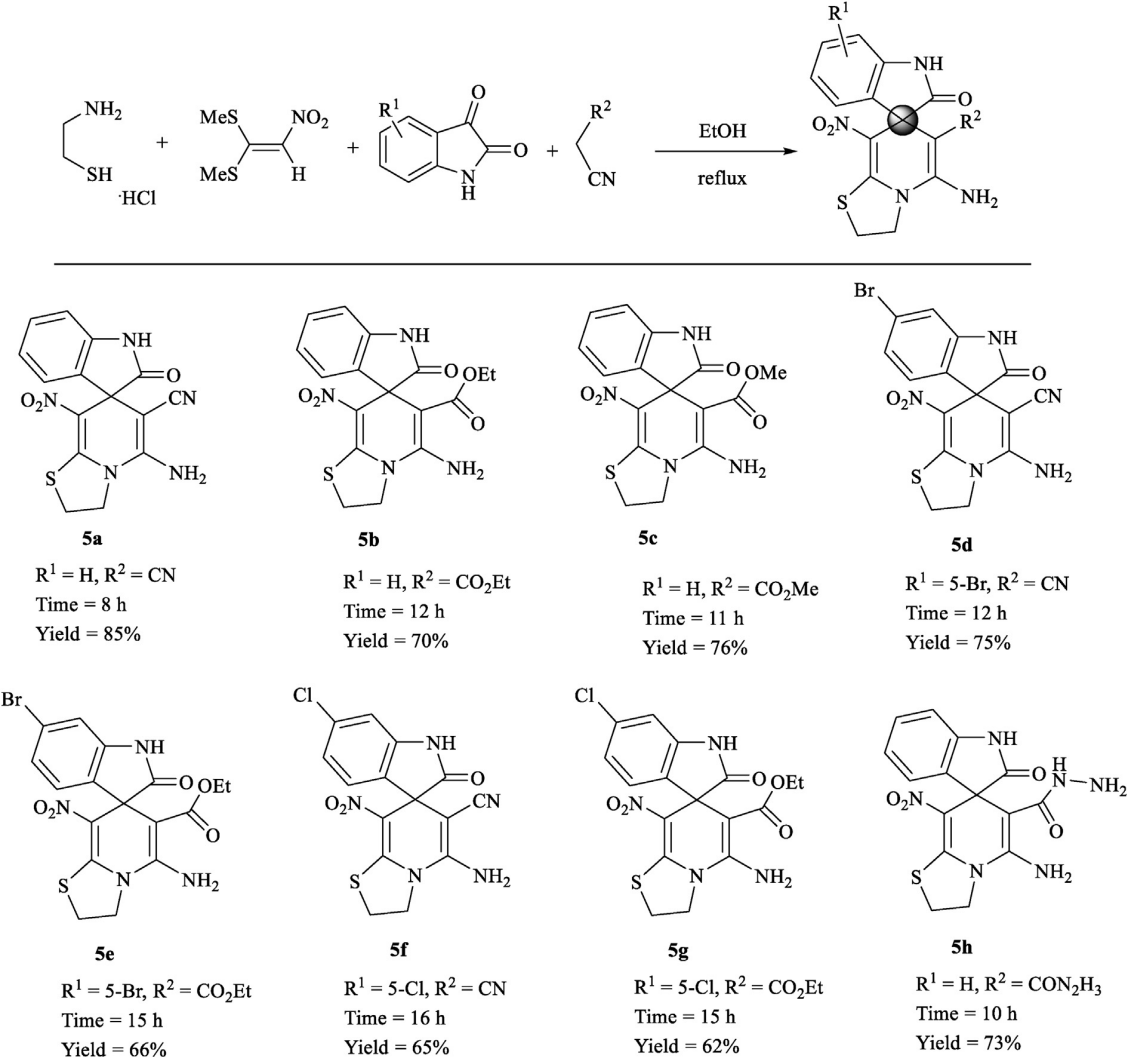
Substrate scope study of functionalized thiazolo pyridine-fused spirooxindoles with a series of isatin and CH-acid compounds.

**Figure 5:**
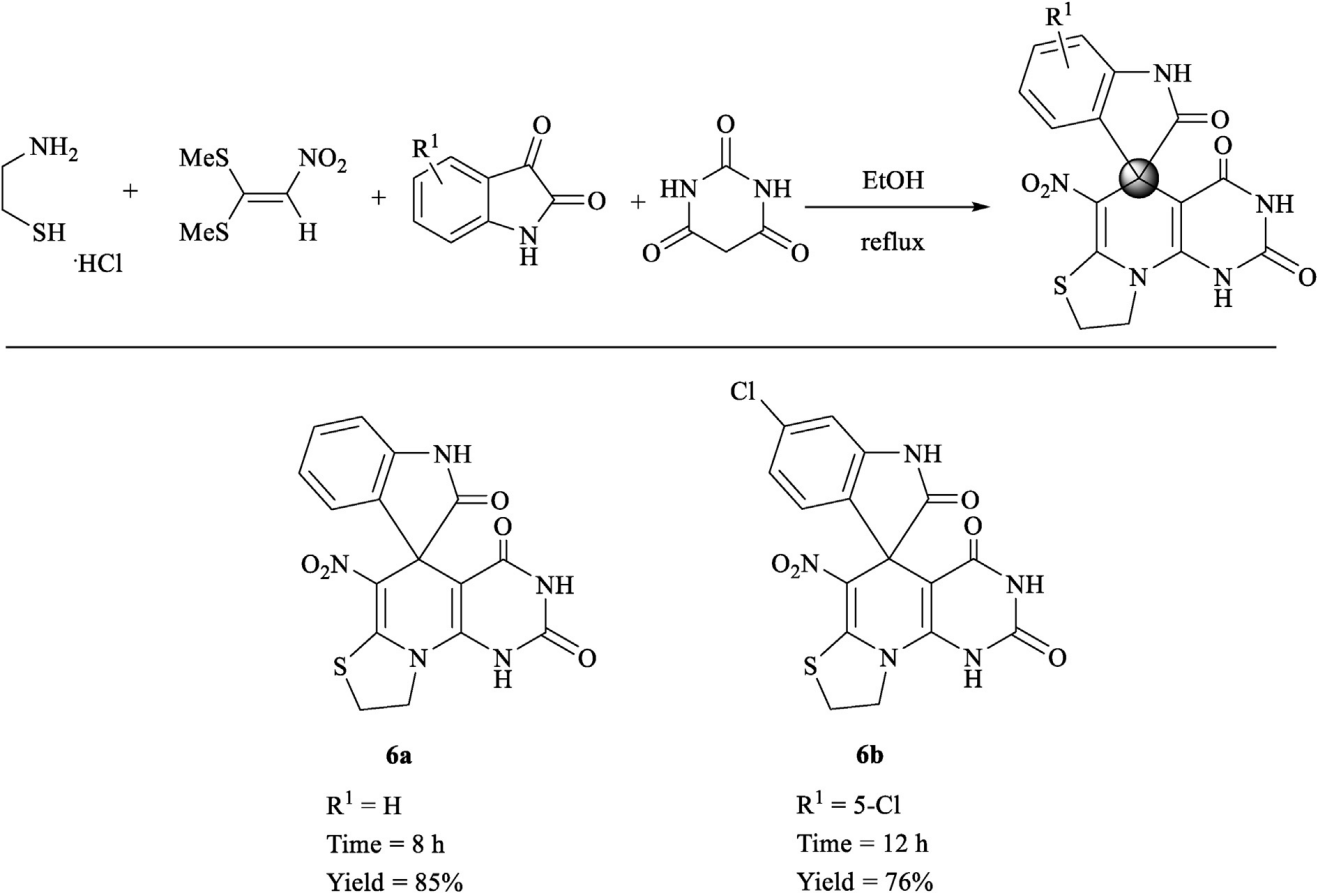
Substrate scope study of functionalized thiazolo pyridopyrimidine-fused spirooxindoles 6 with a series of isatin.

We examined the scope and limitations of this reaction by changing the derivatives of isatin **3**, and the CH-acids **4** and **4′** in the formation of products **5** and **6** (Figures 4 and 5). The reaction continues completely and cleanly using various reagents to provide a new class of spiro products **5** and **6** in 62–85% and 76–85% yields respectively. It is valuable that the product **5** was created in good yield when was applied malononitrile, but when was used ethyl cyanoacetate/methyl cyanoacetate/cyanoacetohydrazide, the product **5** was obtained in longer times and in moderate yields. It is probably due to the low activity of cyanoacetate in comparison of malononitrile. Reactions of cyanoacetamide and cyanothioacetamide did not work well to provide the desired product **5**, because of very less activity. The existence of substituents such as chlorine or bromine on the aromatic ring of isatin affects the process of reaction and provides proposed products in a long time with moderate efficiencies.

The structures of obtained derivatives were confirmed by spectral analysis including FT-IR, ^1^H, ^13^C NMR and mass spectra and also by using elemental analysis (all IR, NMR and mass spectra are illustrated in Supporting Information). The mass spectrum of product **5a** showed a molecular ion peak at *m/z* 341 value, which was in match with the introduced structure. The ^1^H NMR spectrum of **5a** exhibited two multiplets for two CH_2_ groups (*δ* 3.38–3.49, 4.21–4.37 ppm), one singlet for 2H of NH_2_ group (*δ* 6.72 ppm), aromatic region of the spectrum (*δ* 6.78, 6.89–6.94 and 7.14–7.18 ppm) for the aromatic moieties and a broad singlet for the NH group of isatin (*δ* 10.49 ppm). The ^1^H-decoupled ^13^C NMR spectrum of **5a** showed 15 different resonances in agreement with the expected product. Two peaks at *δ* 28.2 and 51.98 ppm for aliphatic hydrocarbons, the specific peak at *δ* 52.2 ppm for C_spiro_, one peak at *δ* 62.2 ppm for carbon attached to the nitrile group, one peak at *δ* 109.8 ppm for carbon connected to the nitro group, the distinguished peak of nitrile was appeared at *δ* 118.5 ppm, two peaks at *δ* 150.0, 160.2 were specified as carbons between sulfur and nitrogen and finally one peak at *δ* 177.5 ppm for one amide carbonyl group confirmed the selective formation of **5a**. The ^1^H NMR spectrum of **6a** displayed two singlets identified for two NH groups of barbituric acid (*δ* 10.42 and 11.00 ppm) and one singlet line for NH group of isatin (*δ* 11.18 ppm). The ^1^H-decoupled ^13^C NMR spectrum of **6a** displayed 16 different resonances. Three peaks at 177.6, 161.1 and 159.6 ppm, which were specified as three amide carbonyl groups and the distinguished peak of C_spiro_ was determined at 51.9 ppm which confirmed the selective formation of **6a**.

## 4 Conclusion

In summary, a novel domino procedure has been developed for the synthesis of spiro thiazole-based heterocycles molecules e.g. diverse thiazolo pyridine-fused spirooxindoles and thiazolo pyridopyrimidine-fused spirooxindoles as attractive synthetic targets. The reactions are easy to carry out simply by adding active methylene compounds, substituted isatin, and 2-nitromethylene thiazolidine as a bifunctional molecule in ethanol. The present method displays attractive advantages such as performing reactions without using any catalyst to remove any transition-metal from the reaction medium, using nano-SiO_2_ as a heterogeneous, recyclable, low toxicity and inexpensive promoter, simple workup procedure and formation of highly functionalized molecules with a drug-like structure. It is expected that more valuable heterocycles synthesized *via* subsequent developments of this procedure.

## Declarations

### Author contribution statement

Shima Nasri: Conceived and designed the experiments; Analyzed and interpreted the data; Wrote the paper.

Mohammad Bayat: Conceived and designed the experiments; Analyzed and interpreted the data; Contributed reagents, materials, analysis tools or data.

Hassan Vasheghani Farahani, Solmaz Karami: Performed the experiments.

### Funding statement

This work was supported by Iran National Science Foundation (98004758).

### Competing interest statement

The authors declare no conflict of interest.

### Additional information

No additional information is available for this paper.

## Appendix A Supplementary data

Revised Supporting Information R2 Revised Supporting Information R2
