# Supplementary material for: Synthesis of new functionalized thiazolo pyridine-fused and thiazolo pyridopyrimidine-fused spirooxindoles via one-pot reactions
Source: Heliyon. 2020 Mar 31;6(3):e03687. doi: 10.1016/j.heliyon.2020.e03687 (PMC7114753; doi:10.1016/j.heliyon.2020.e03687)
Supplement: Revised Supporting Information R2 [file mmc1.doc]

**Supporting Information**

**Synthesis of new functionalized thiazolo pyridine-fused and thiazolo pyridopyrimidine-fused spirooxindoles *via* one-pot reactions**

Shima Nasri, Mohammad Bayat*, Hassan Vasheghani Farahani, Solmaz Karami

*Department of Chemistry, Imam Khomeini International University, Qazvin, Iran.*

*bayat_mo@yahoo.com*

**The Table of Contents**

| **Title** | **Page** |
| --- | --- |
| Title, author’s name, address and table of contents | 1 |
| Structure of all products **5a-h** and **6a-b** | 2 |
| ^1^H and ^13^C NMR and IR and Mass spectra of **5a** | 3-6 |
| ^1^H and ^13^C NMR and IR and Mass spectra of **5b** | 7-10 |
| ^1^H and ^13^C NMR and Mass spectra of **5c** | 11-13 |
| ^1^H and ^13^C NMR and Mass spectra of **5d** | 14-16 |
| ^1^H and ^13^C NMR and IR and Mass spectra of **5e** | 17-20 |
| ^1^H and ^13^C NMR spectra of **5f** | 21-22 |
| ^1^H and ^13^C NMR spectra of **5g** | 23-24 |
| ^1^H NMR and IR and Mass spectra of **5h** | 25-27 |
| ^1^H and ^13^C NMR and Mass spectra of **6a** | 28-30 |
| ^1^H NMR and Mass spectra of **6b** | 31-32 |

Structure of all products **5a-h** and **6a-b**

**
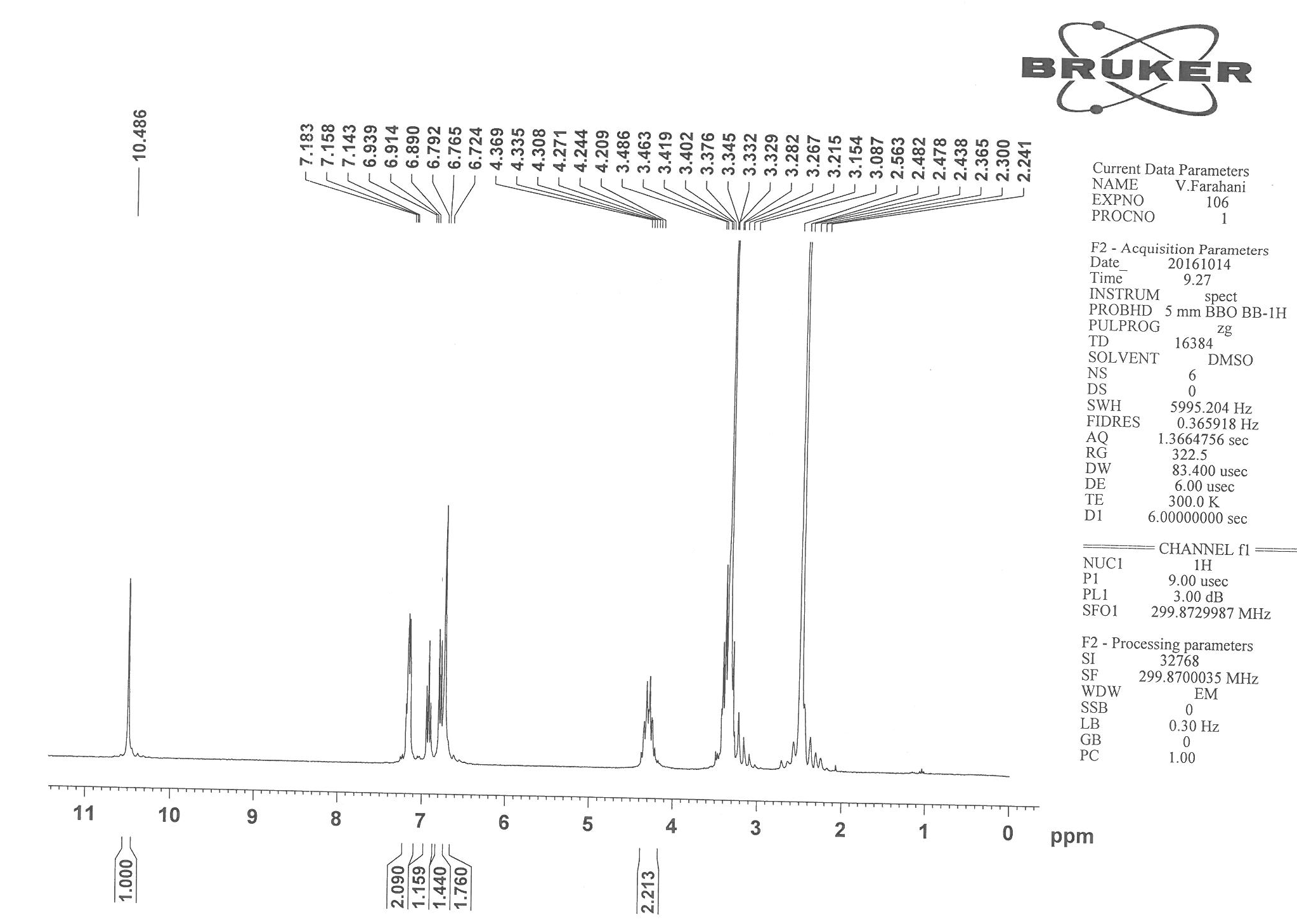
**

**^1^H NMR of 5a**


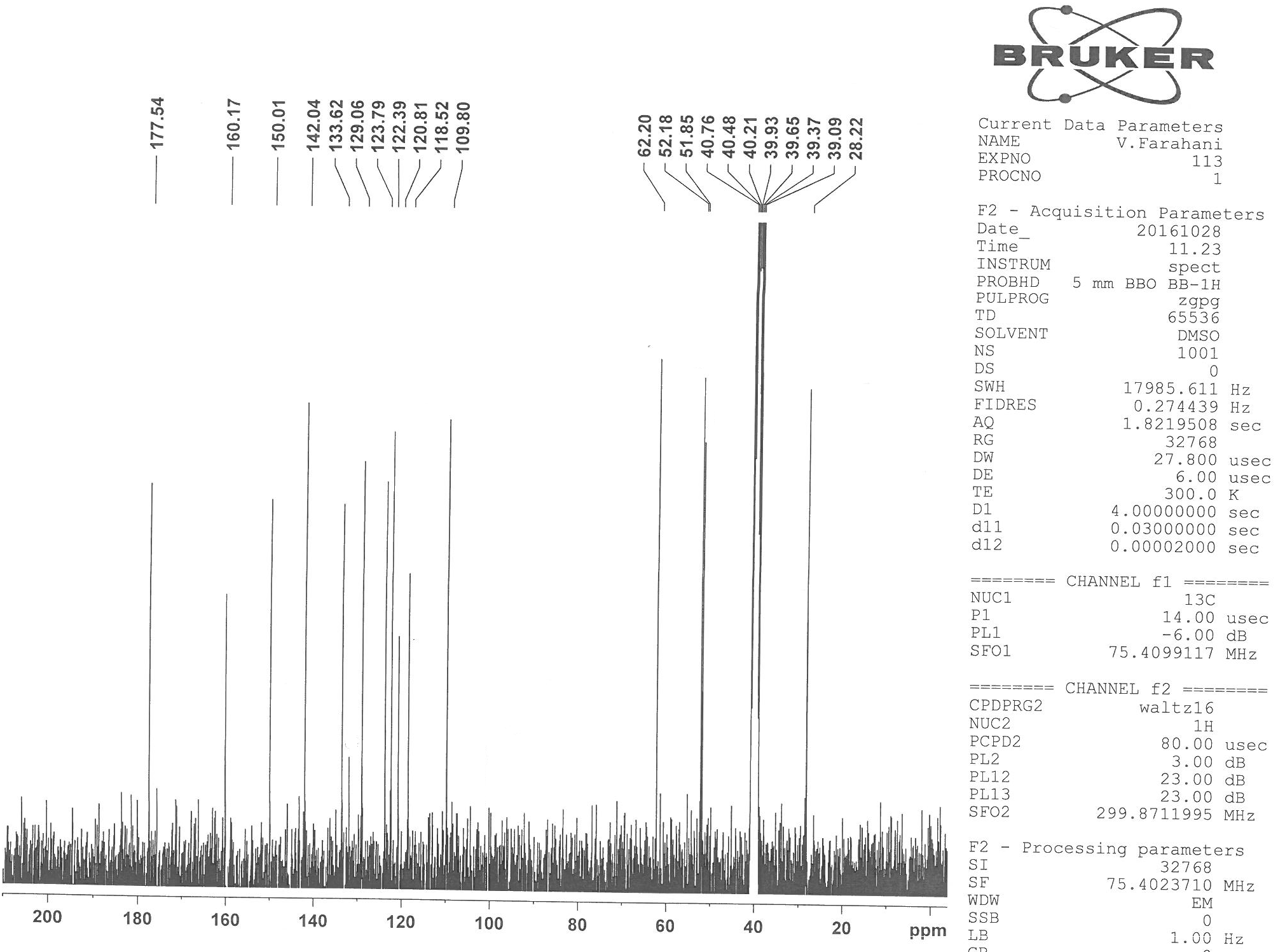


**^13^C NMR of 5a**


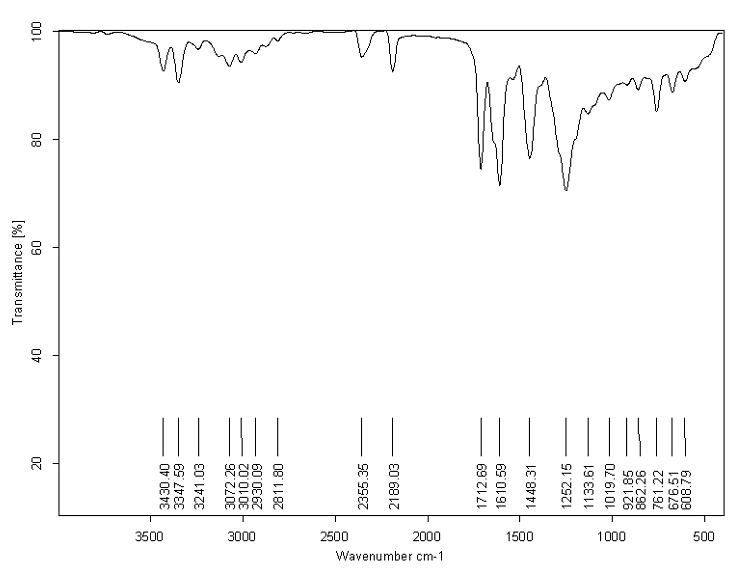


**IR of 5a**


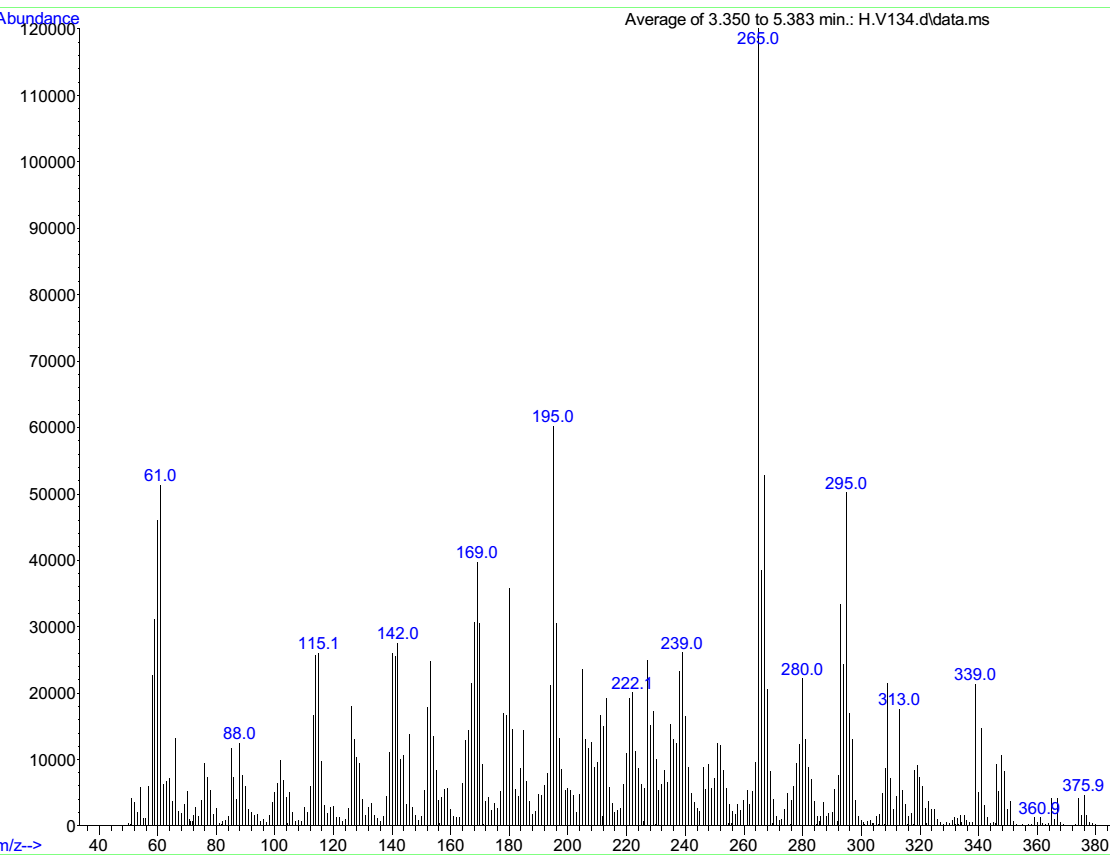


**Mass of 5a**

**
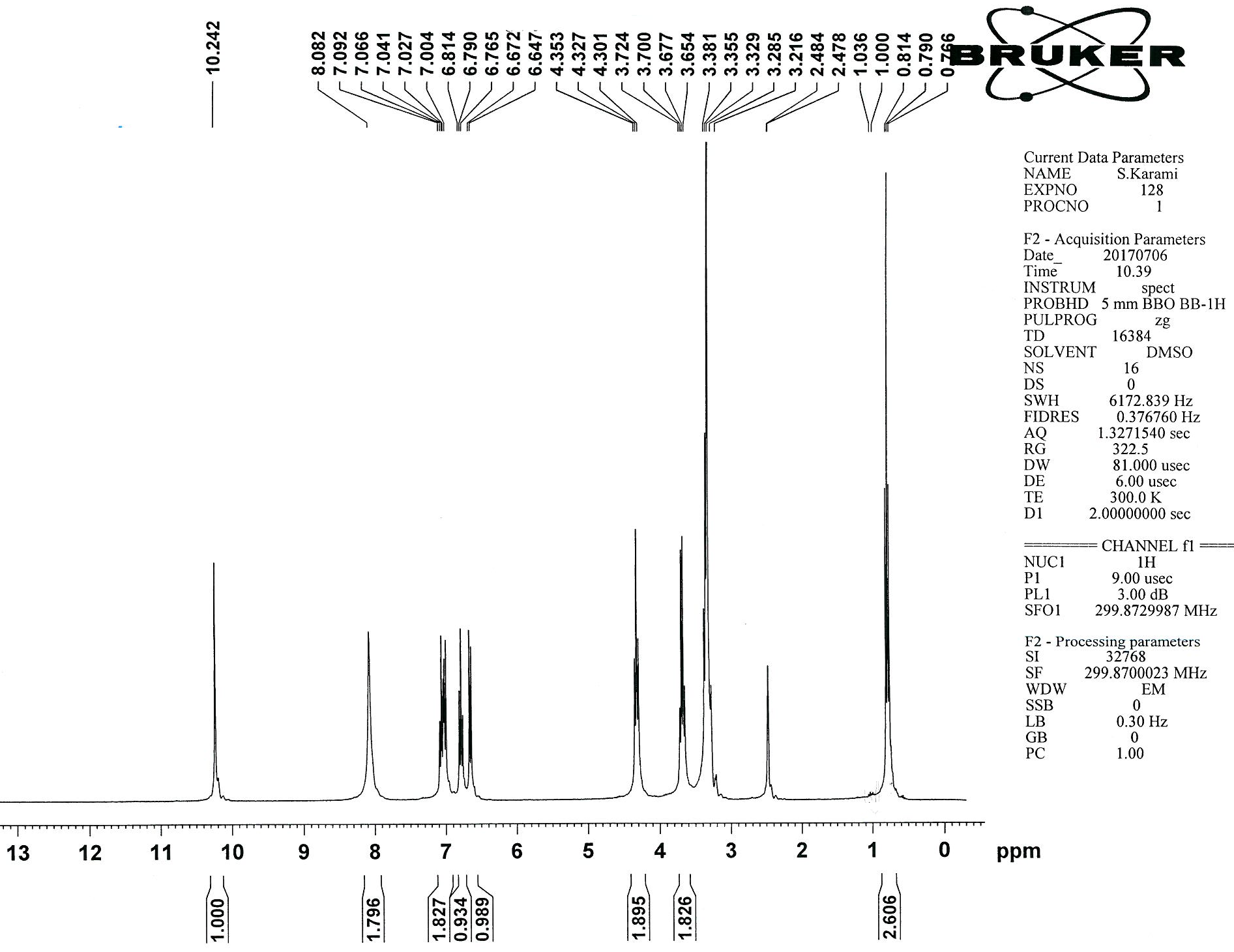
**

**^1^H NMR of 5b**


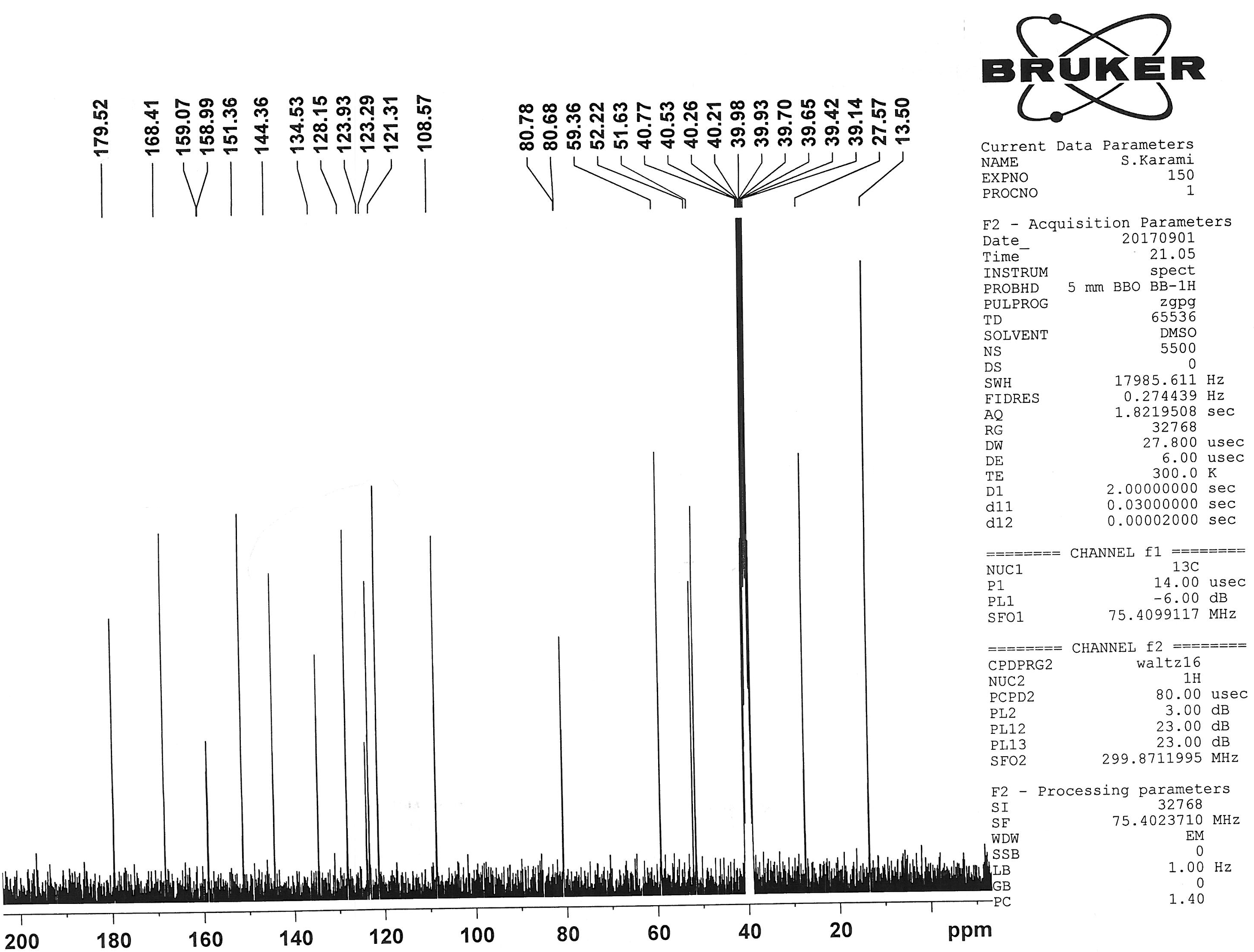


**^13^C NMR of 5b**


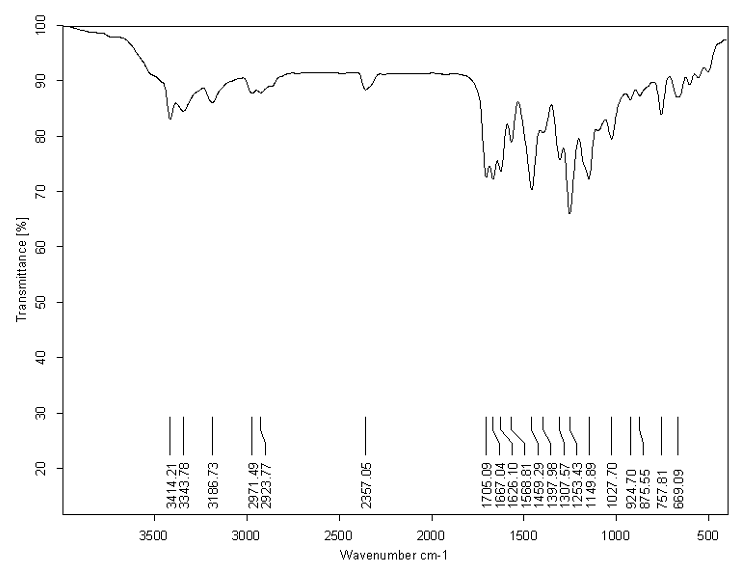


**IR of 5b**


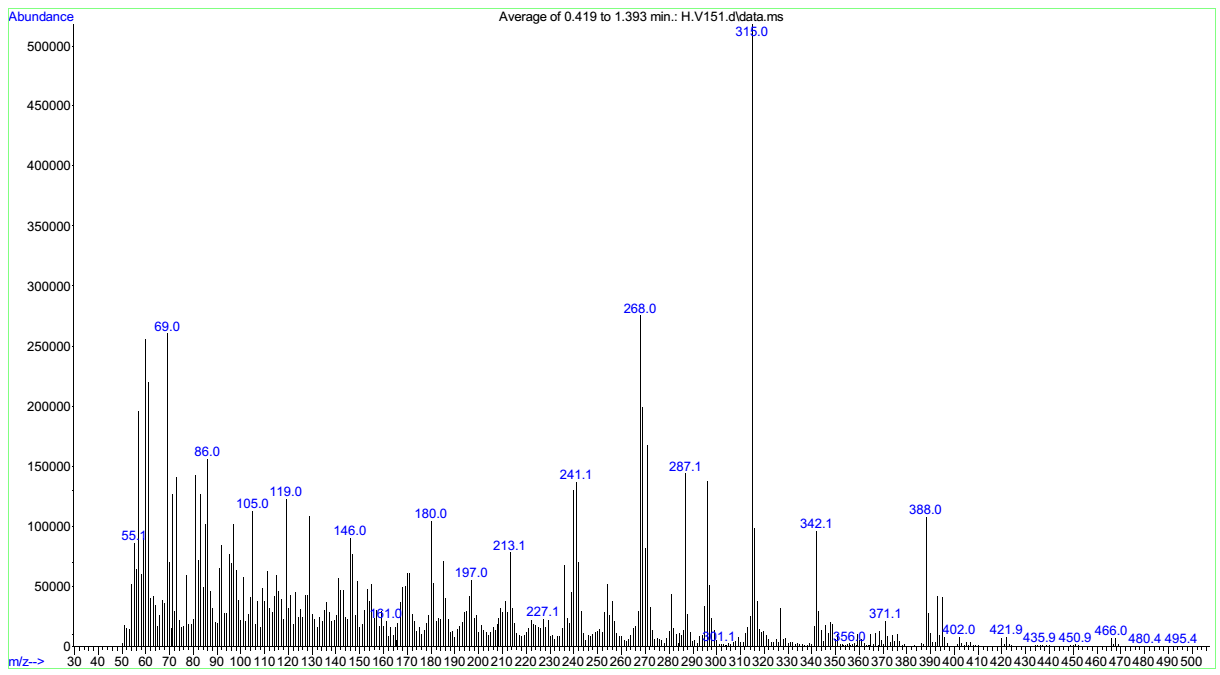


**Mass of 5b**


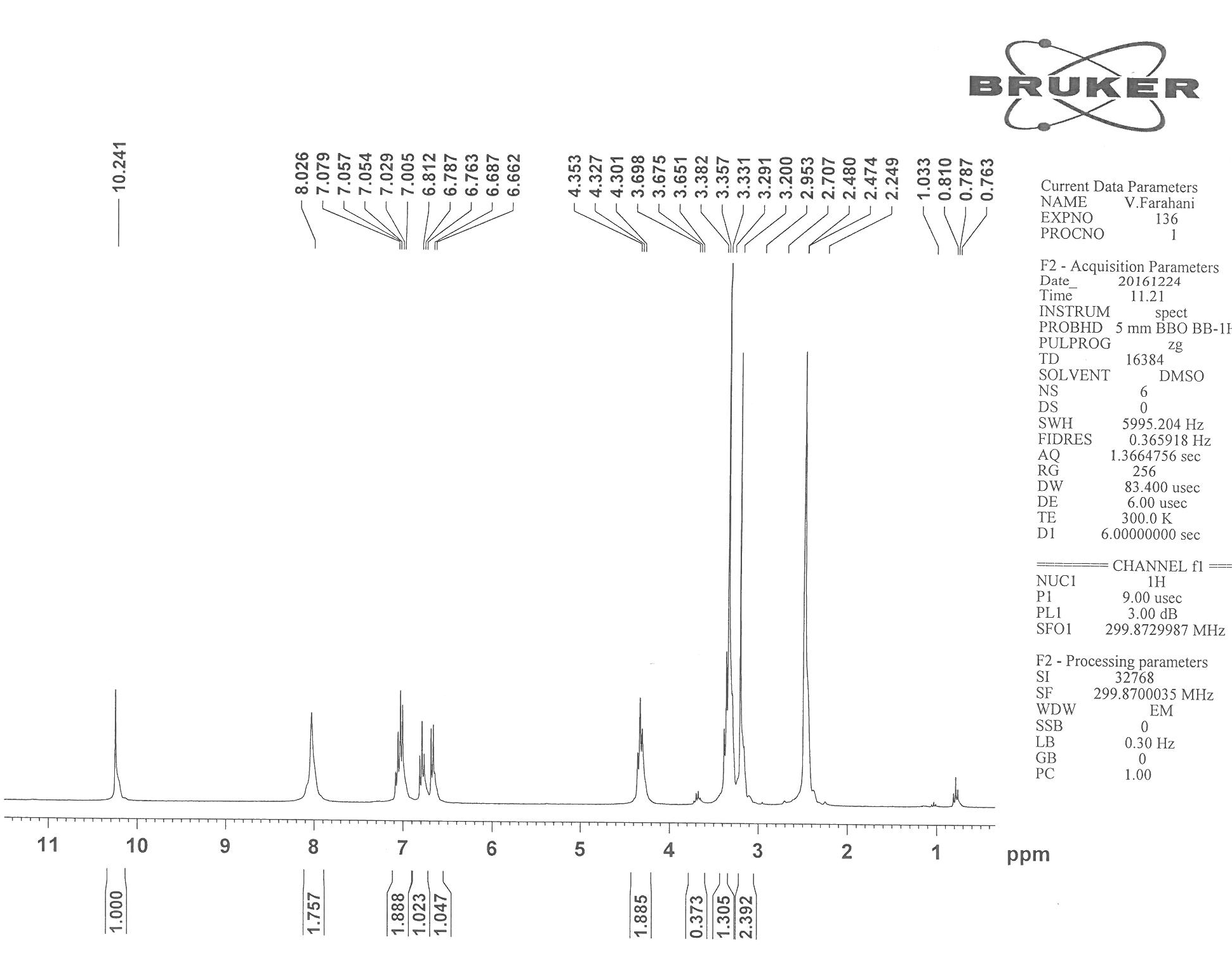


**^1^H NMR of 5c**


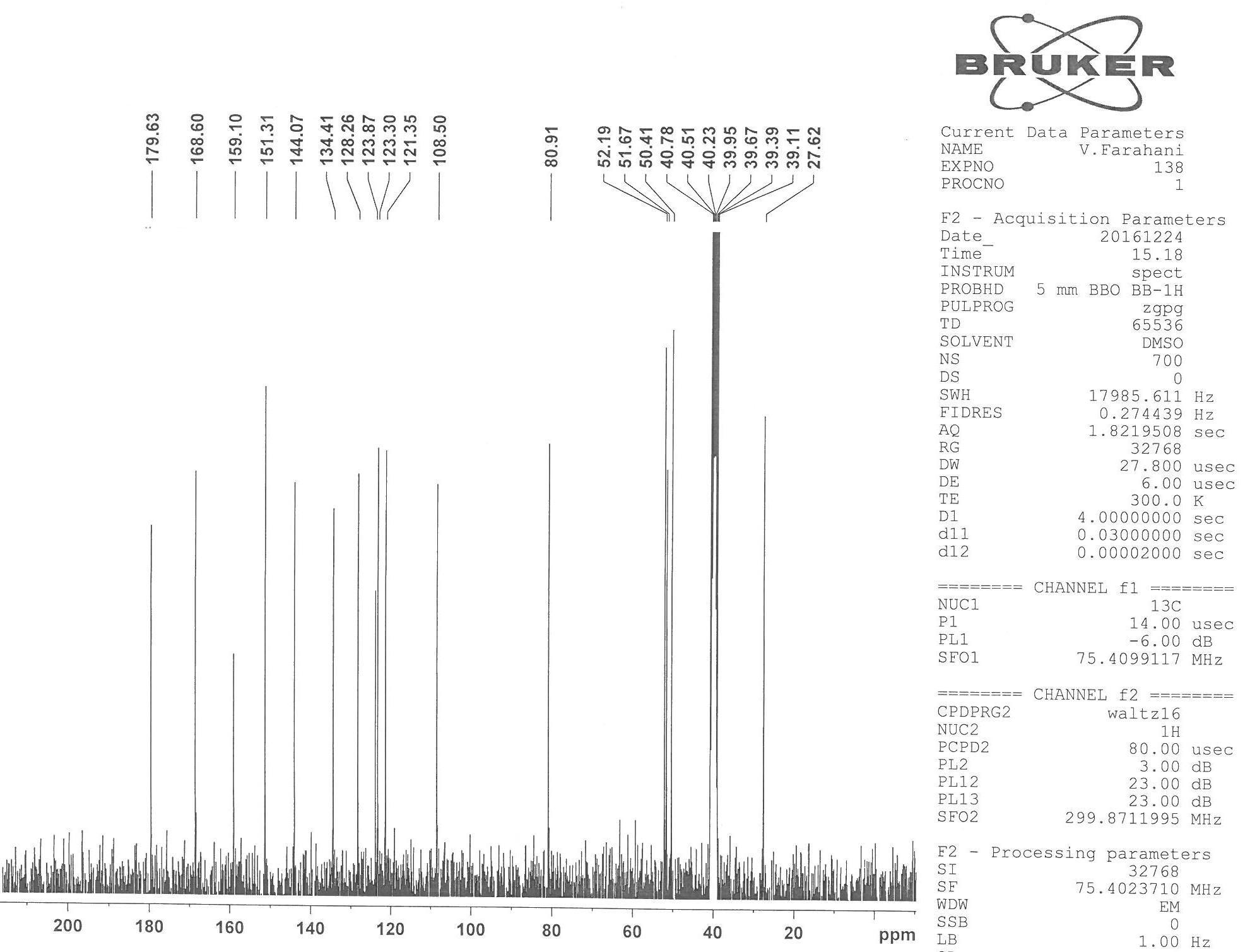


**^13^C NMR of 5c**


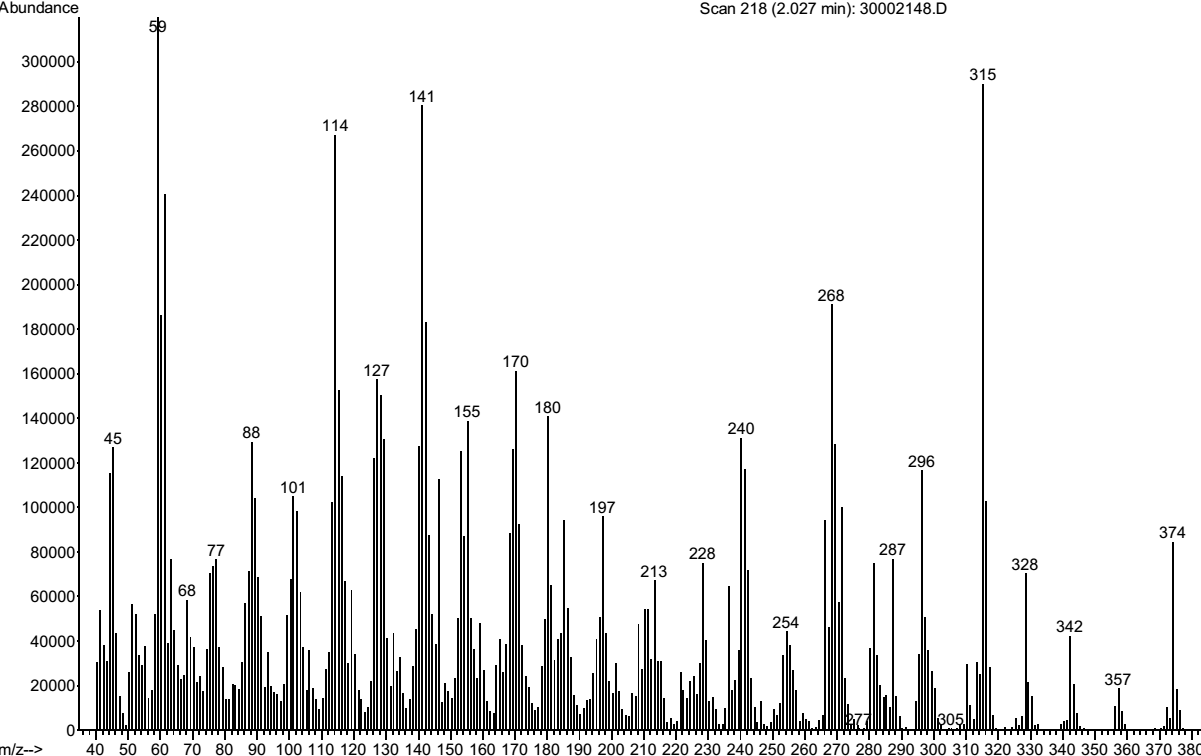


**Mass of 5c**


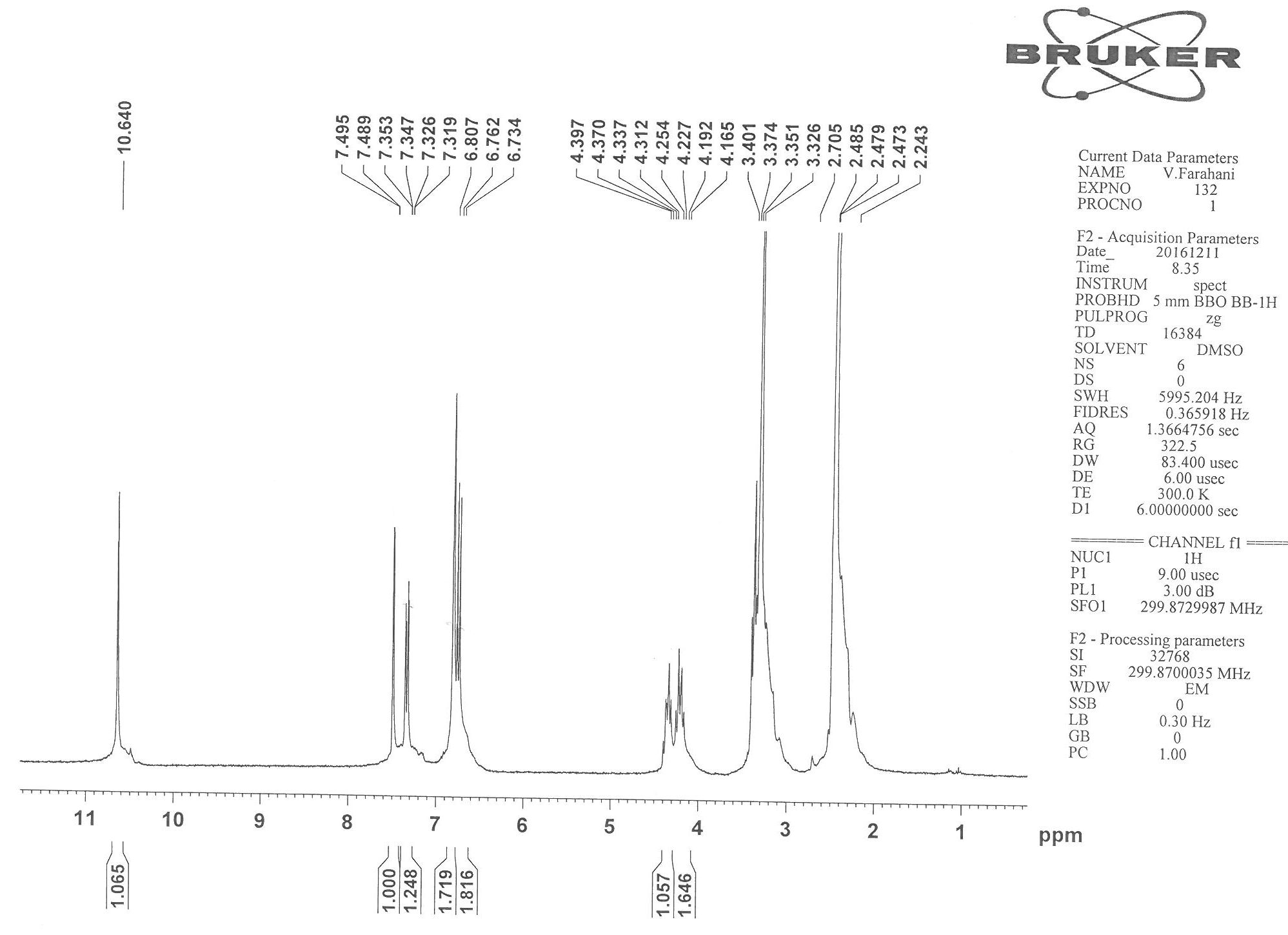


**^1^H NMR of 5d**


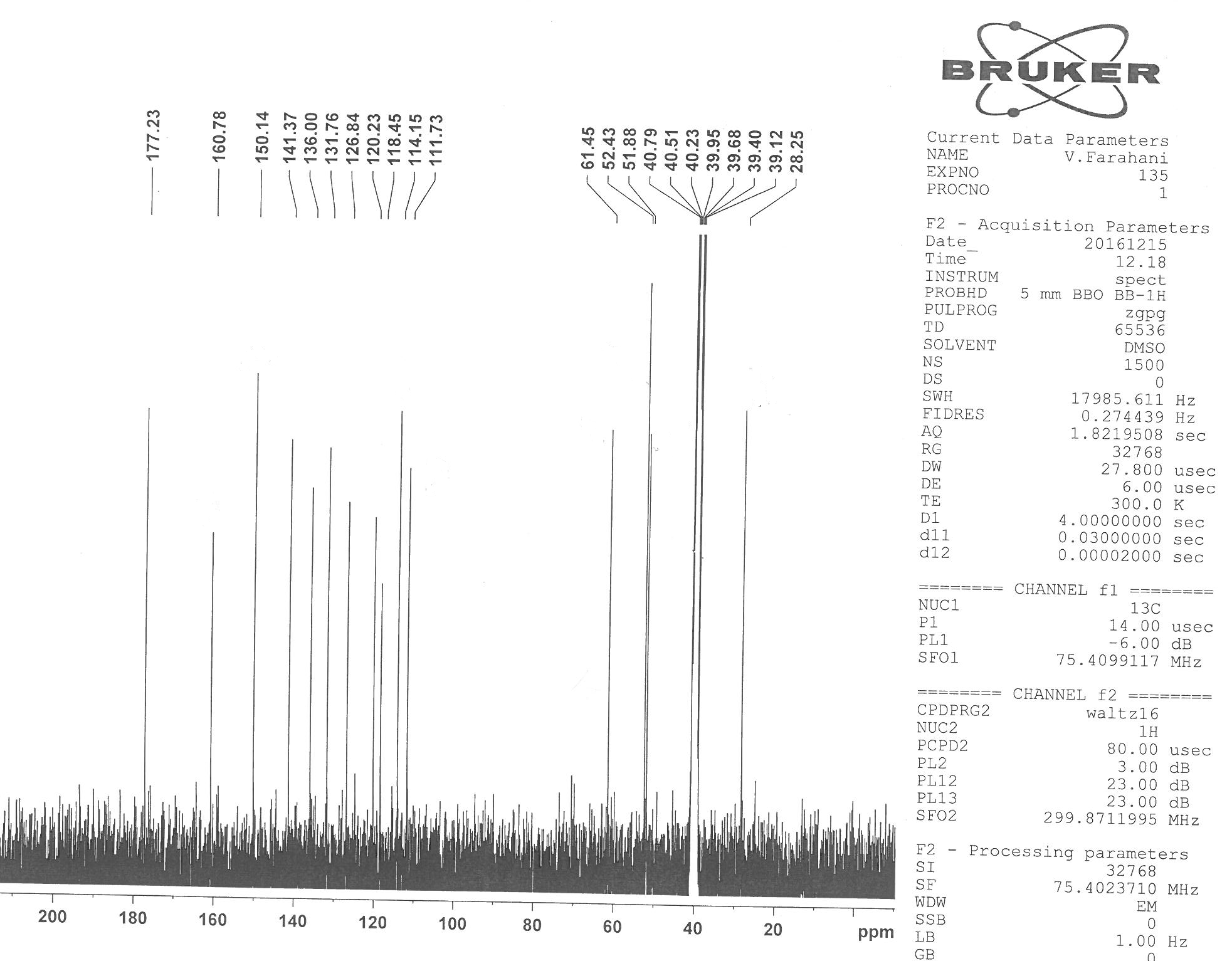


**^13^C NMR of 5d**


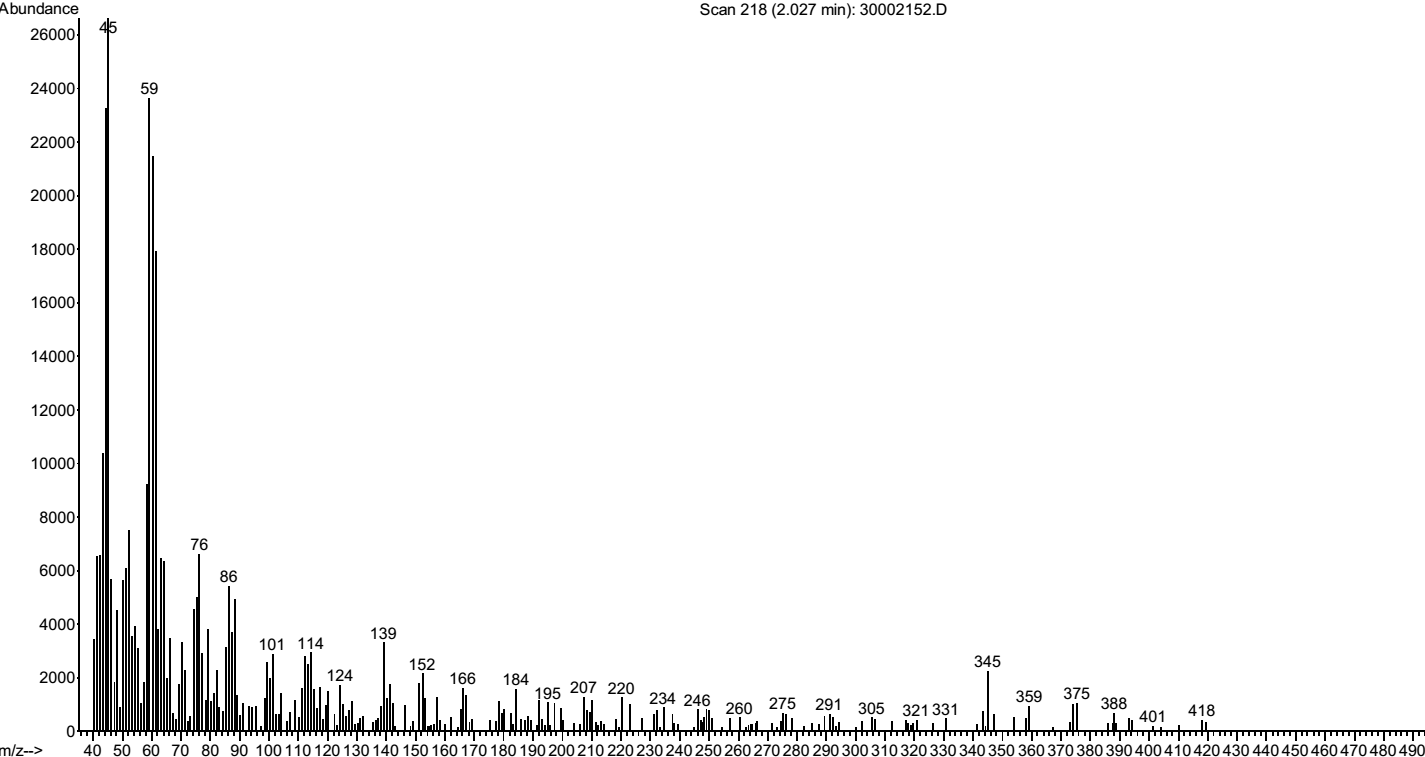


**Mass of 5d**


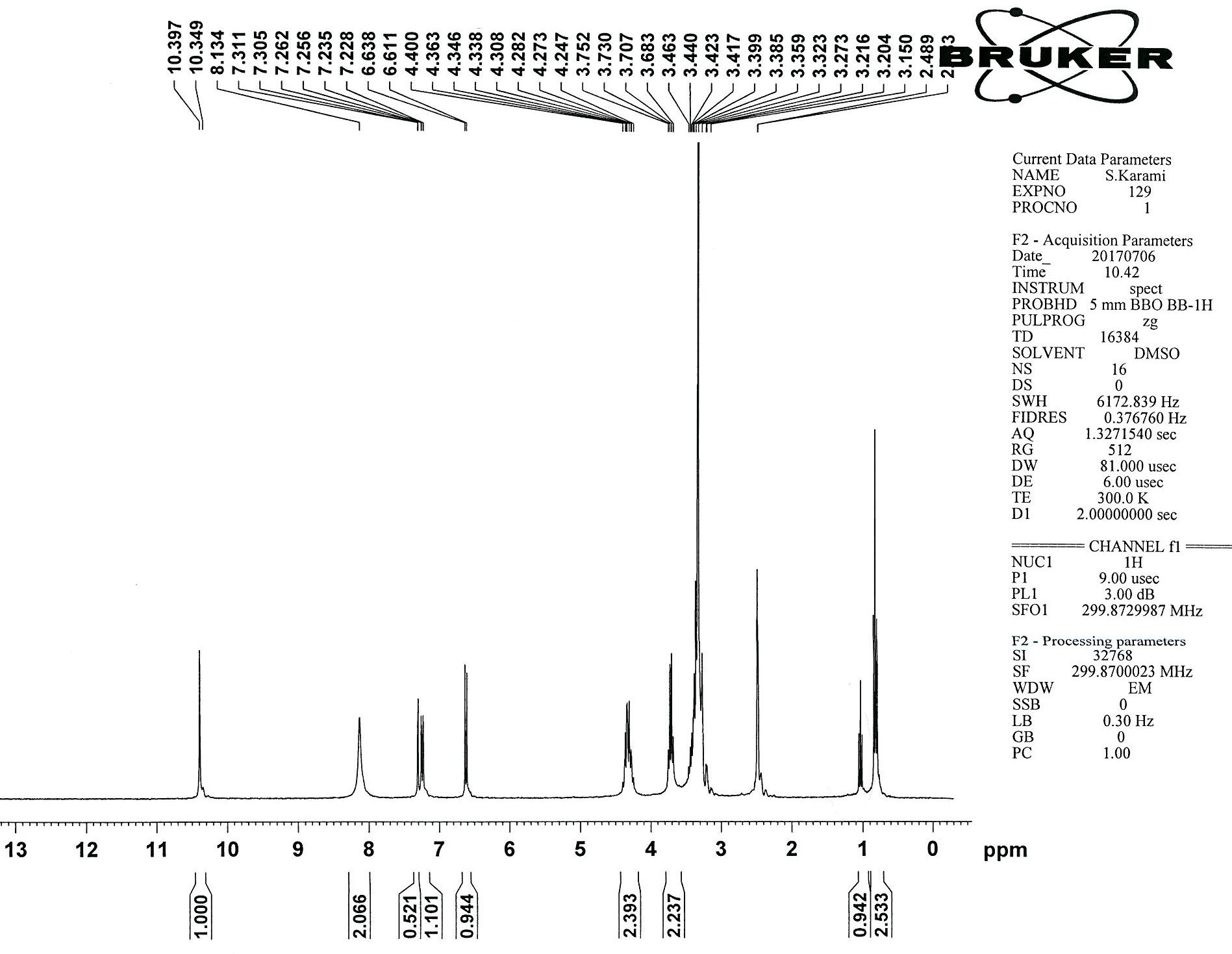


**^1^H NMR of 5e**


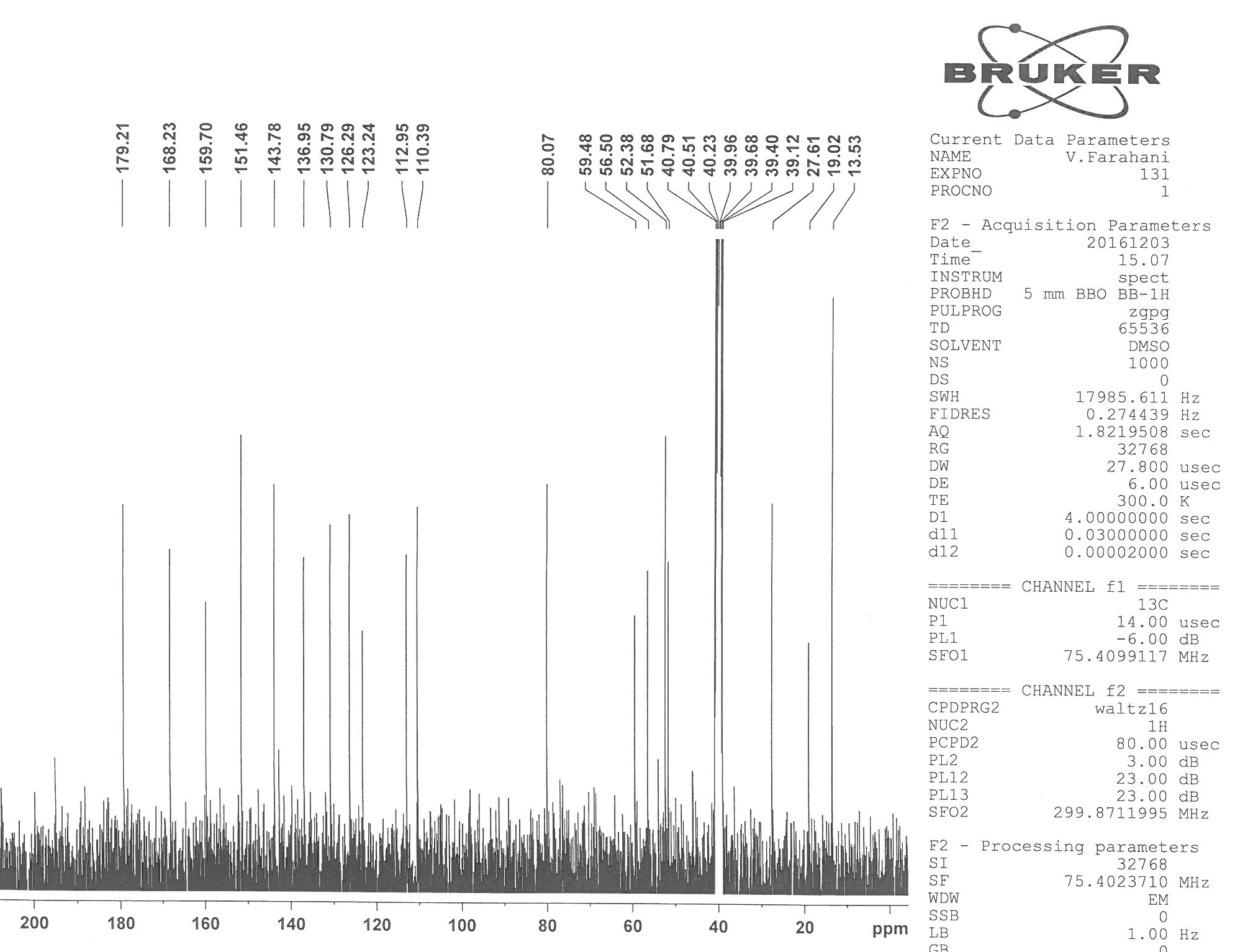


**^13^C NMR of 5e**


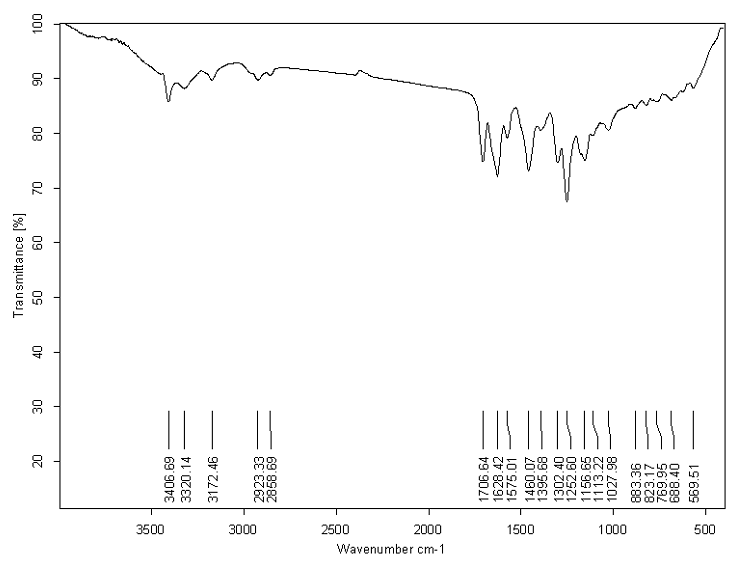


**IR of 5e**


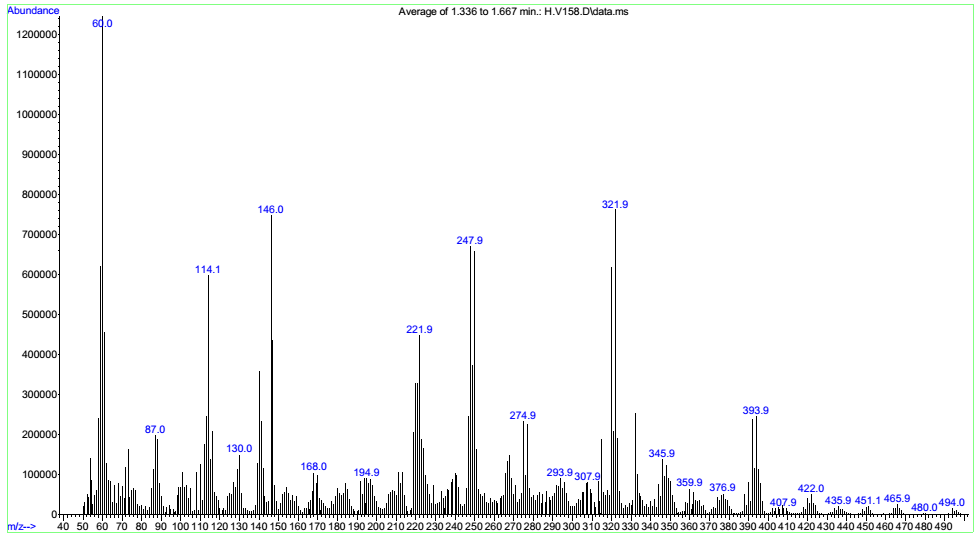


**Mass of 5e**

**
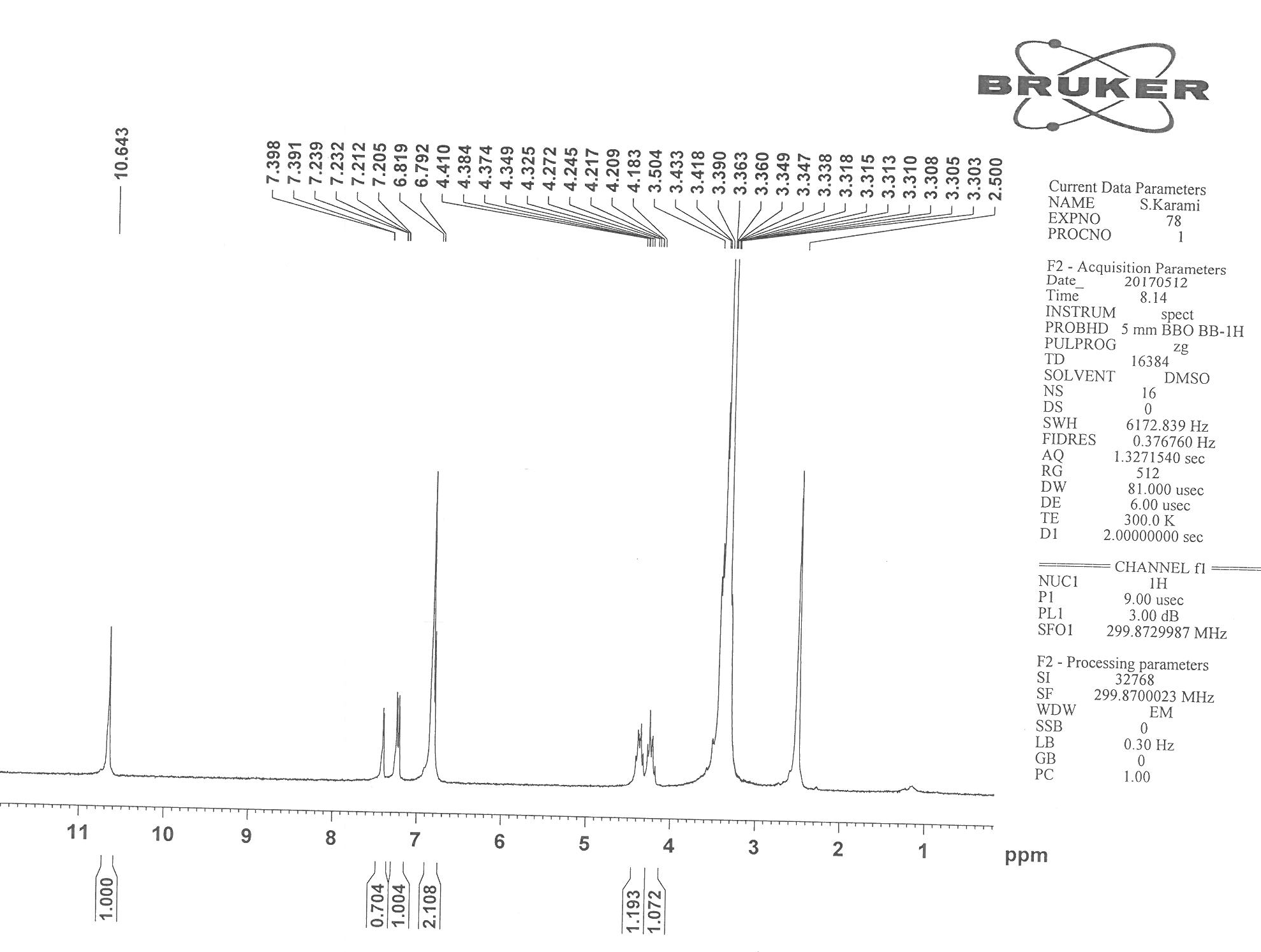
**

**^1^H NMR of 5f**

**
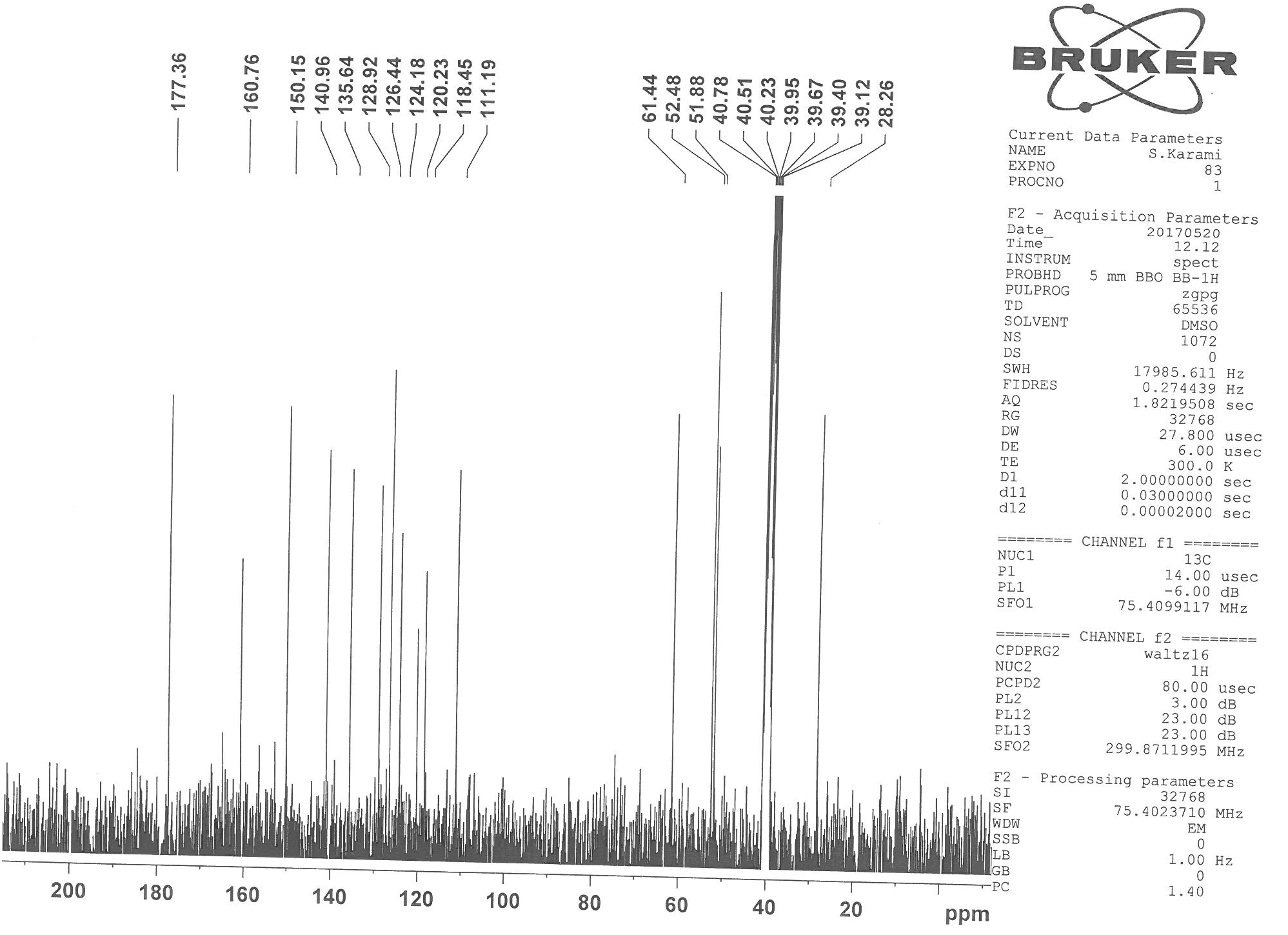
**

**^13^C NMR of 5f**

**
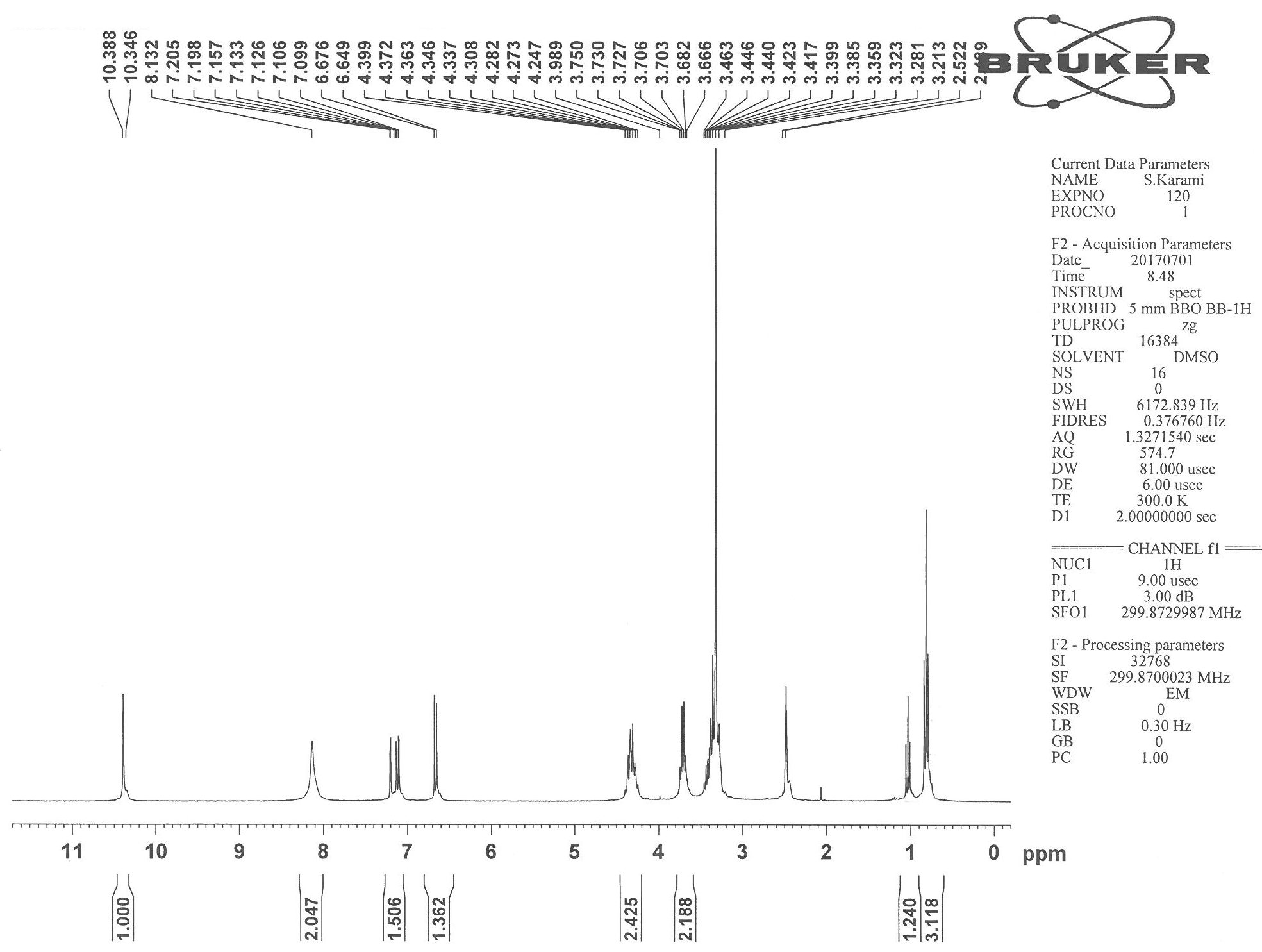
**

**^1^H NMR of 5g**

**
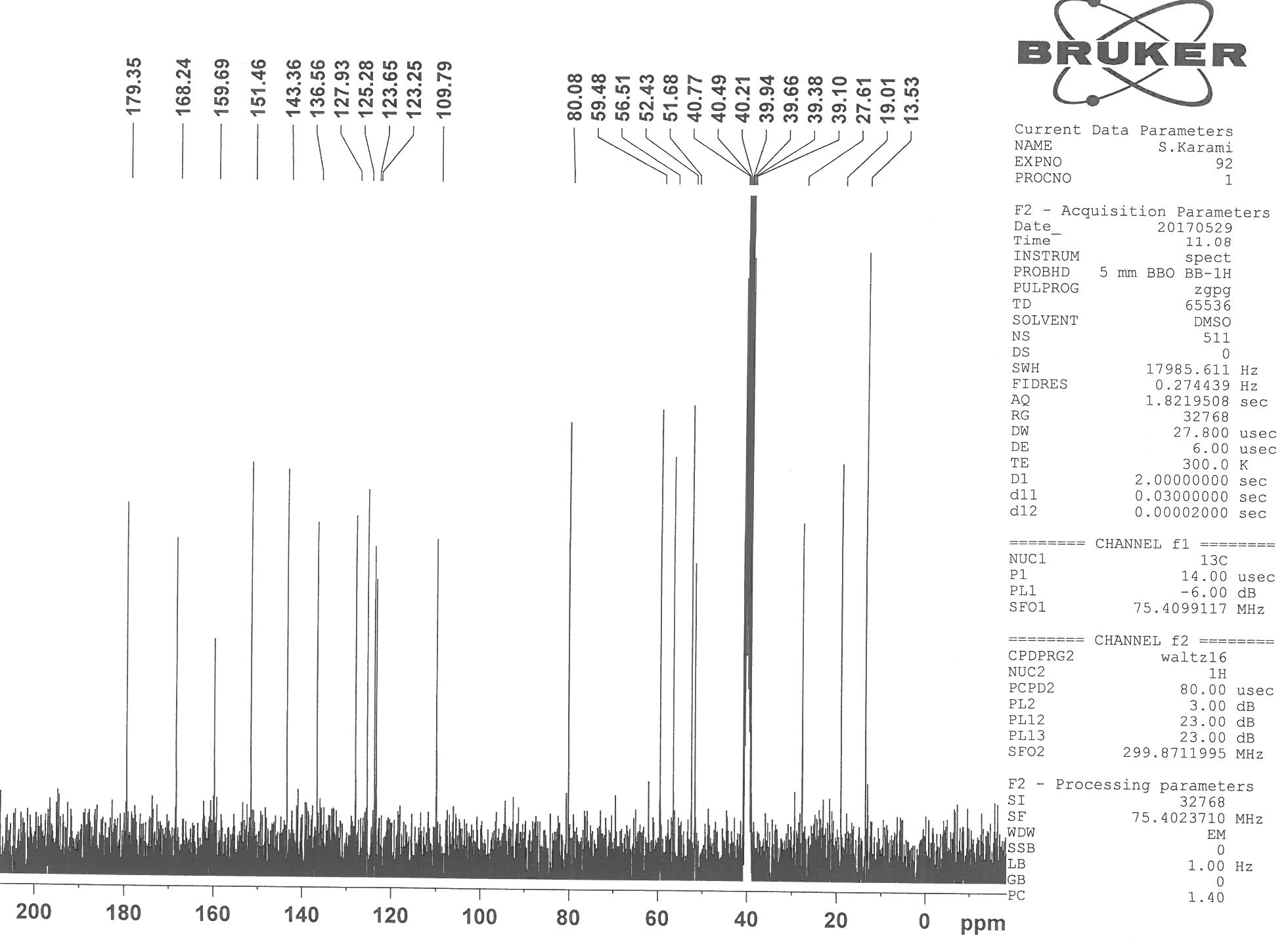
**

**^13^C NMR of 5g**


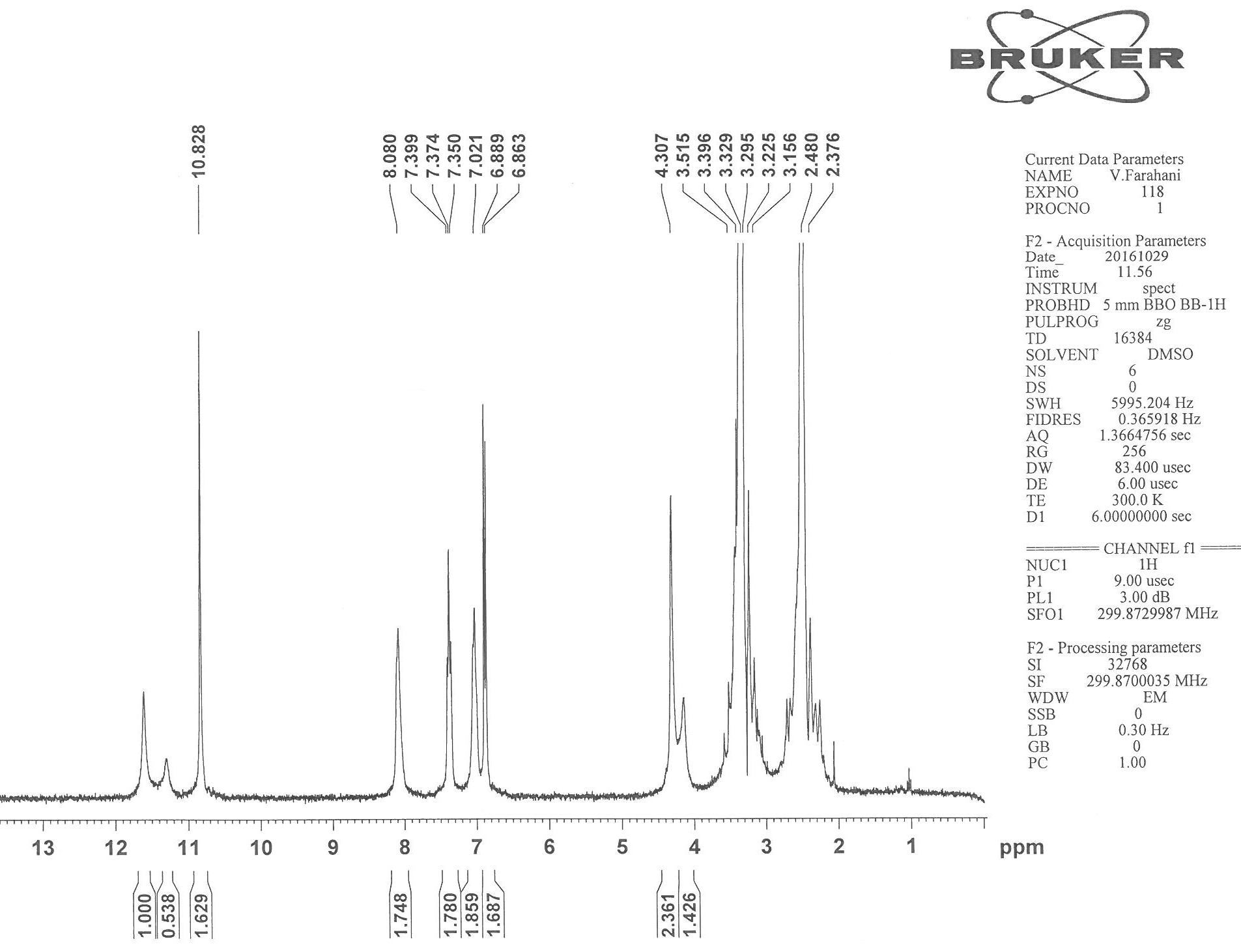


**^1^H NMR of 5h**


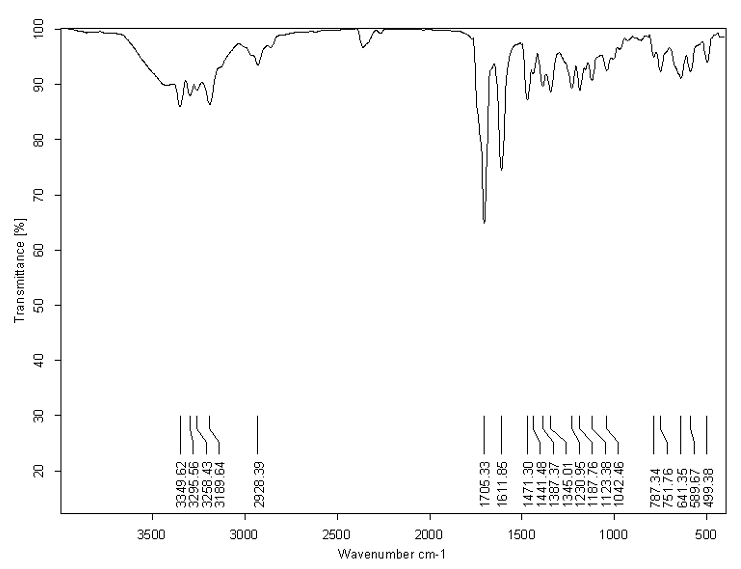


**IR of 5h**


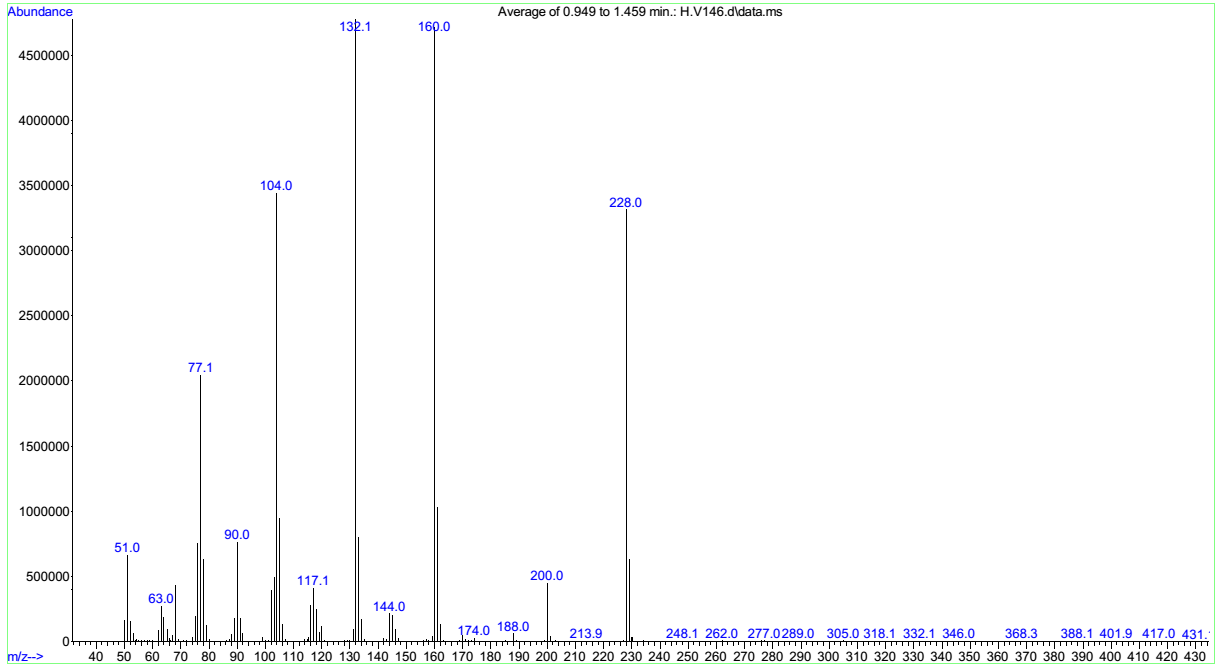


**Mass of 5h**

**
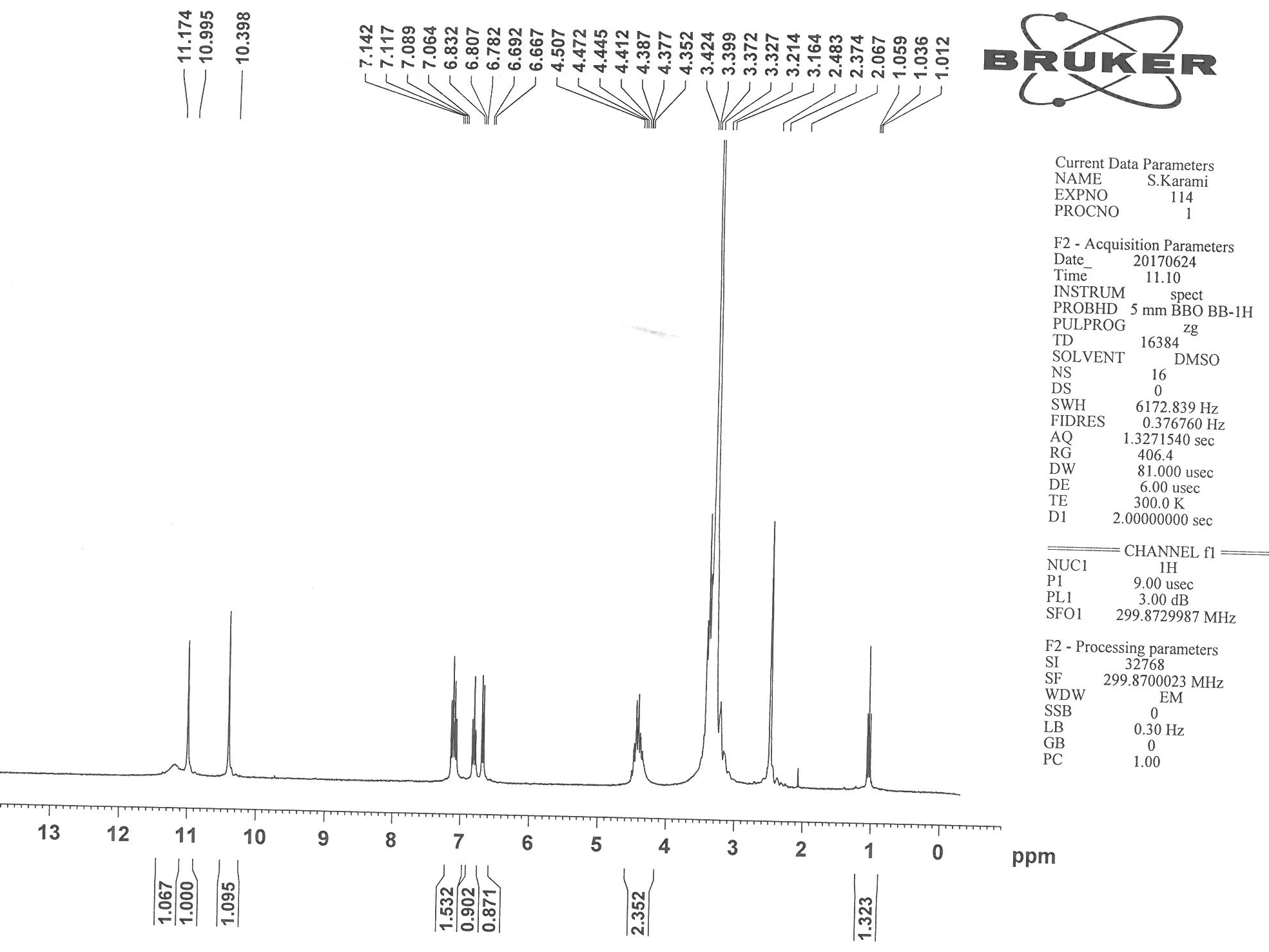
**

**^1^H NMR of 6a**


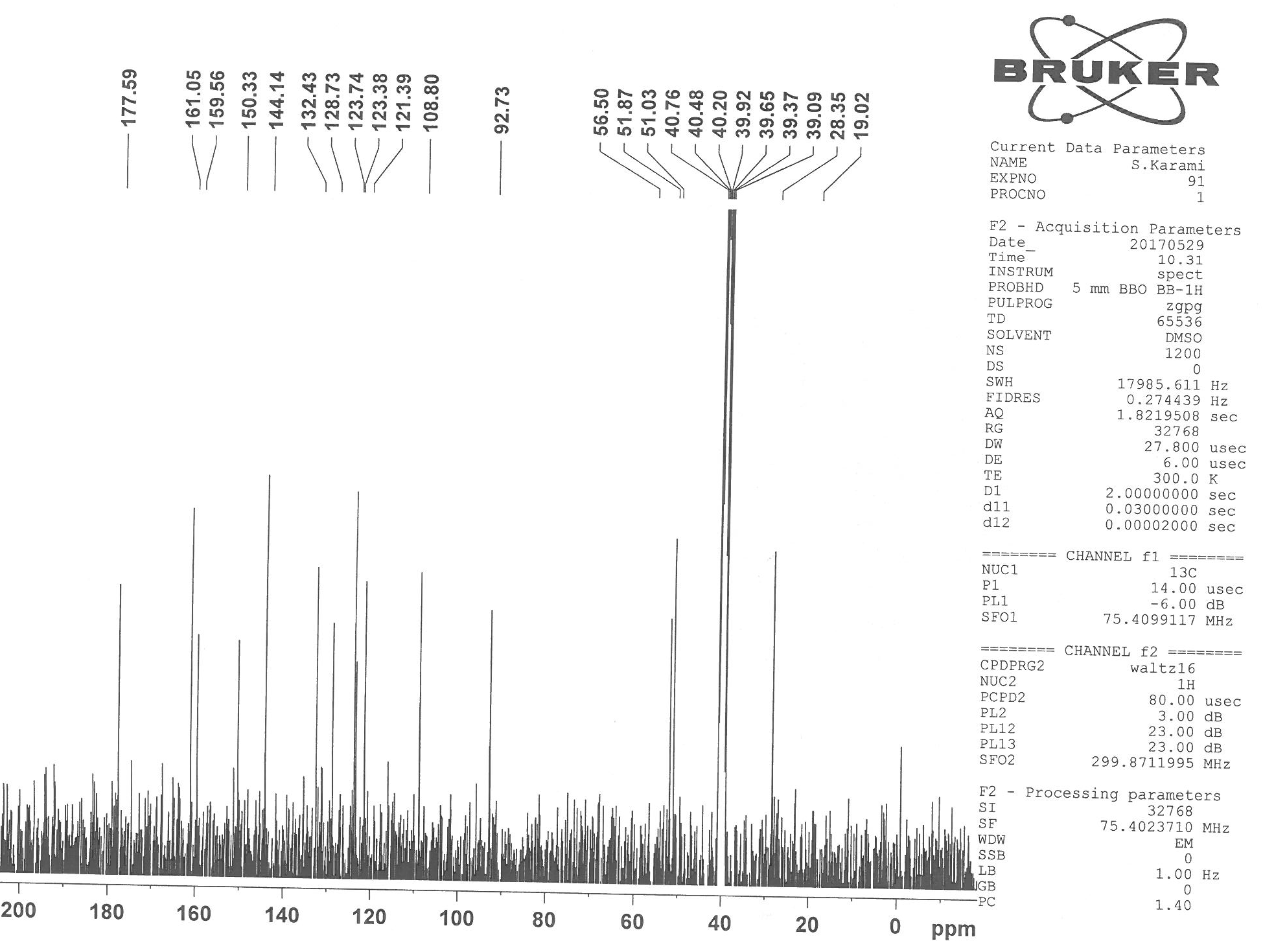


**^13^C NMR of 6a**


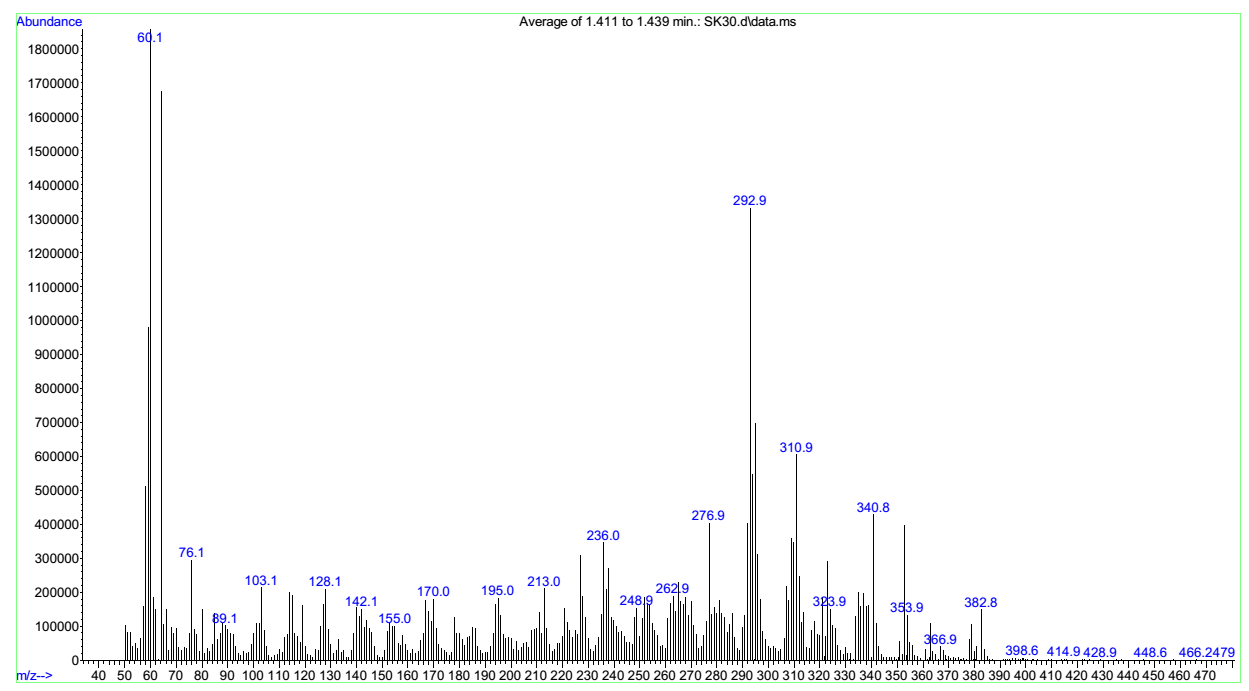


**Mass of 6a**


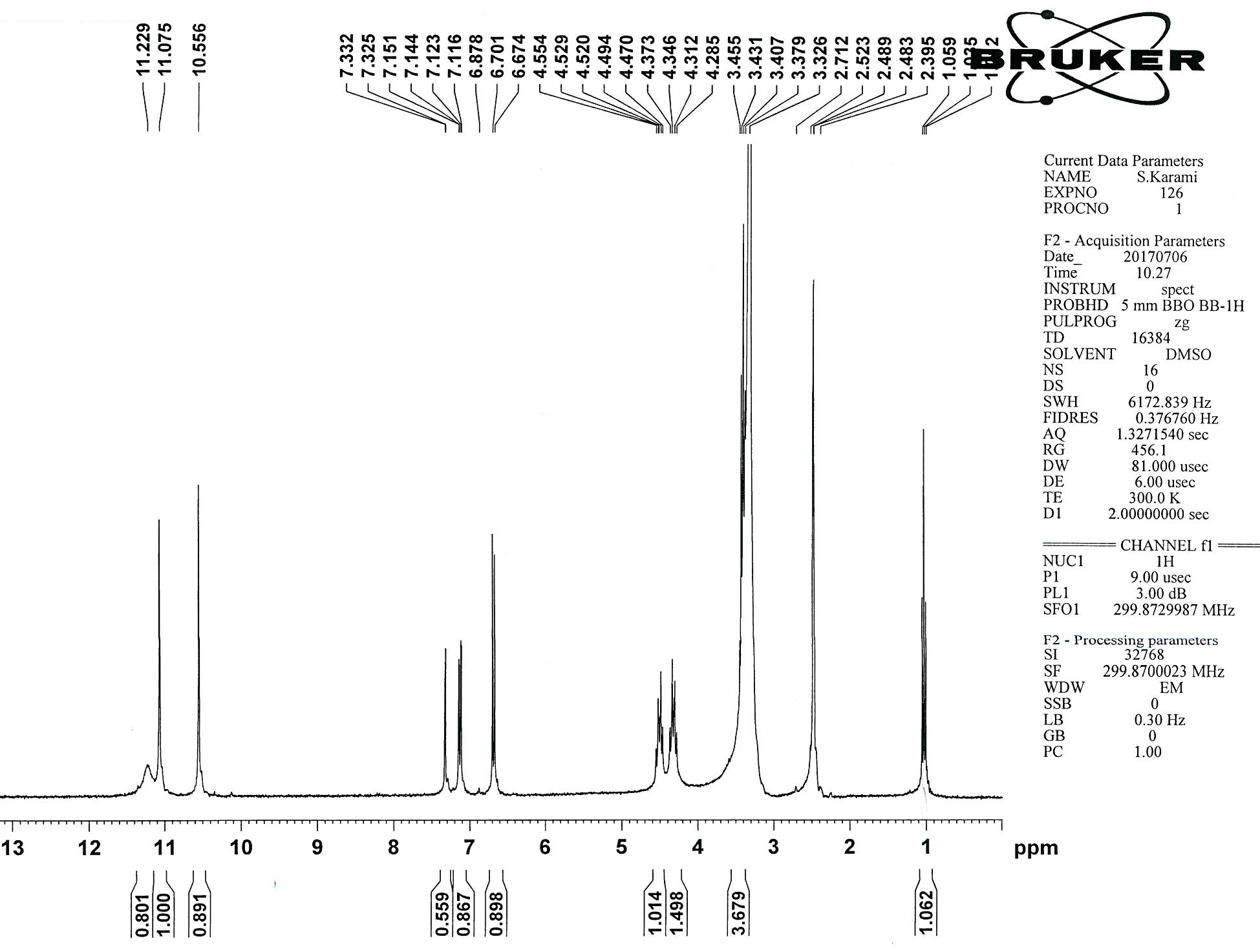


**^1^H NMR of 6b**


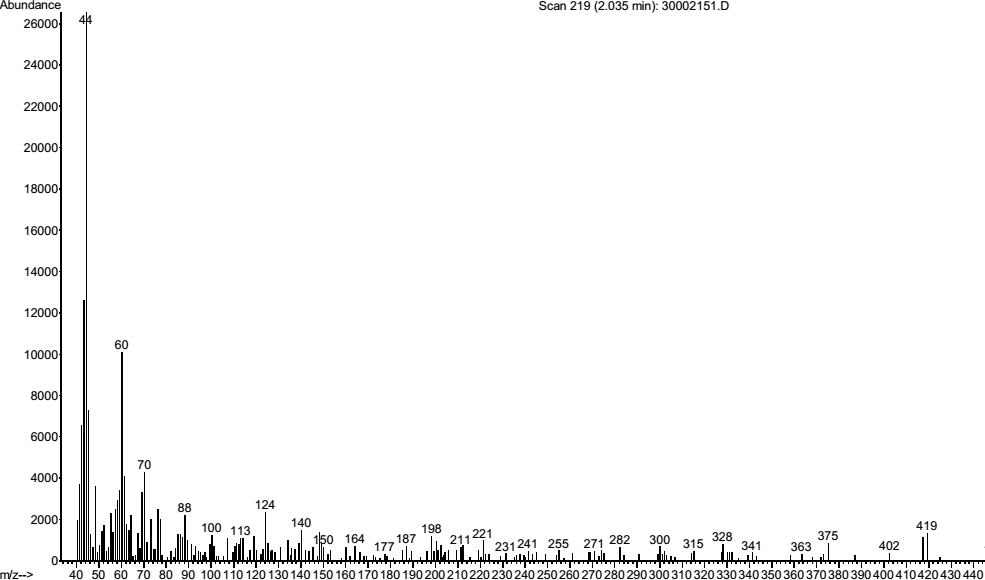


**Mass of 6b**
